# Targeting prooxidant MnSOD effect inhibits triple-negative breast cancer (TNBC) progression and M2 macrophage functions under the oncogenic stress

**DOI:** 10.1038/s41419-021-04486-x

**Published:** 2022-01-11

**Authors:** Aushia Tanzih Al Haq, Hong-Yu Tseng, Li-Mei Chen, Chien-Chia Wang, Hsin-Ling Hsu

**Affiliations:** 1 Institute of Molecular and Genomic Medicine, National Health Research Institutes, Miaoli, Taiwan; 2 Department of Life Sciences, National Central University, Taoyuan, Taiwan

**Keywords:** Cancer microenvironment, Breast cancer, Oncogenes

## Abstract

Triple-negative breast cancer (TNBC) has been shown with high mitochondrial oxidative phosphorylation and production of reactive oxygen species (ROS). MnSOD (*SOD2*) is a mitochondrial antioxidant defense that has been implicated in inhibition of human malignancies. However, the impact of MnSOD on immunosuppressive macrophage functions and TNBC aggressiveness has never been explored. We found here that *SOD2*^high^ is primarily observed in the aggressive subtypes of HER2(+) breast cancers and TNBCs patients. Further analyses demonstrated that the oncoprotein multiple copies in T-cell malignancy-1 (MCT-1 or *MCTS1*) induces mitochondrial superoxide dismutase (MnSOD) in TNBC cells by stabilizing the transcription factor Nrf2. *SOD2*^high^/*MCTS1*^high^ expression correlates with a poor prognosis in breast cancer patients. MnSOD in TNBC cells functions as a prooxidant peroxidase that increases mitochondrial ROS (mROS) and adaptation to oxidative stress under the oncogenic effect. Interleukin-6 (IL-6) in the MCT-1 pathway elevates Nrf2/MnSOD and mROS levels. Knockdown of MnSOD inhibits TNBC cell invasion, breast cancer stem cells (BCSCs), mROS, and IL-6 excretion promoted by MCT-1. TNBC cells deficient in MnSOD prevent the polarization and chemotaxis of M2 macrophages but improve the ability of M1 macrophages to engulf cancer cells. Quenching mROS with MitoQ, a mitochondria-targeted non-metal-based antioxidant MnSOD mimics, effectively suppresses BCSCs and M2 macrophage invasion exacerbated by MnSOD and MCT-1. Consistently, silencing MnSOD impedes TNBC progression and intratumoral M2 macrophage infiltration. We revealed a novel stratagem for TNBC management involving targeting the MCT-1 oncogene-induced mitochondrial prooxidant MnSOD pathway, which prevents the development of an immunosuppressive tumor microenvironment.

## Introduction

Triple-negative breast cancers (TNBCs) are characterized by a lack of hormone receptors for estrogen (ER), progesterone (PR), and human epidermal growth factor 2 (HER2) [1]. Patients with TNBC not only have a poor prognosis and high incidences of relapse and metastasis [2], but also frequently develop drug resistance [1, 3]. Finding novel targetable biological features or therapeutic regimens for TNBCs thus become a high priority.

Cancer aggressiveness is reinforced by the interaction between tumor and host immune cells that secrete protumorigenic chemokines to support the development of an immunosuppressive tumor microenvironment (TME) [4]. Tumor-associated macrophages (TAMs) are the predominant immune cells infiltrating solid tumors [5, 6]. TAMs are recruited to and differentiate in primary or metastatic sites, depending on the inflammatory cytokines or chemokines secreted by tumoral or stromal cells [6]. TAMs are mainly classified as inflammatory M1 and protumorigenic M2 macrophages. M1 macrophages act as innate host defense with tumoricidal functions via the production pro-inflammatory cytokines [4]. Conversely, M2 macrophages secrete anti-inflammatory cytokines and highly express immunosuppressive receptors, which enhance tumor growth and immune escape [4–6].

Oxidative stress creates intrinsic insults in carcinogenesis by inducing genetic instability, activating growth factor-dependent pathways, and disrupting aerobic metabolism [7]. To mitigate the impact of mitochondrial reactive oxygen species (mROS), mitochondrial superoxide dismutase (MnSOD or *SOD2*) controls superoxide radical anions (O_2_.^−^) derived from electron transport chain byproduct and converts O_2_.^−^ into the diffusible strong oxidant H_2_O_2_ [8]. Since elevated ROS cause cancer [9], increased MnSOD are expected to suppress tumors. However, contrary to this expectation, recent studies have shown that MnSOD promotes signaling cascades that support malignant transformation [10], cell survival [11], and cancer stemness capacity [12], which overtake its oxidative defense mechanism. In multiple myeloma cells, MnSOD inhibits the binding of the transcription factor AP-1 to regulate proinflammatory cytokines interleukin 6 (IL-6 or *IL6*) [13]. The IL-6 promoter contains an antioxidant response element (ARE) [14], thus suggesting crosstalk between IL-6 and ROS scavenger activity, such as MnSOD. Nevertheless, the impact of MnSOD on tumor immunity remains unclear.

Multiple copies in T-cell lymphoma-1 (MCT-1), also known as malignant T-cell-amplified sequence 1 (*MCTS1*), is a pro-oncoprotein that regulates the translation reinitiation [15] and recycles post-termination ribosomal subunits [16] through complexes with density-regulated proteins [17] and the 40 S ribosomal subunit [18]. MCT-1 protects A549 lung cancer cells from oxidative damage by inducing the YY1/EGFR/MnSOD axis [19]. We recently reported that MCT-1 stimulates IL-6 secretion, which advances epithelial-mesenchymal transition (EMT) progression [20], TNBC stemness, and M2 macrophage plasticity.

Further elucidating the role of the MCT-1/MnSOD axis in the oxidative TME, we now verify that MCT-1 shifts the antioxidant role of MnSOD toward a prooxidant effect via IL-6 signaling that elevates mROS in aggressive TNBC cells, which enhances M2 macrophage functions and TNBC expansion.

## Results

### Enhancement of MCT-1 and MnSOD is a poor prognostic marker in aggressive breast cancer

**Fig. 1:**
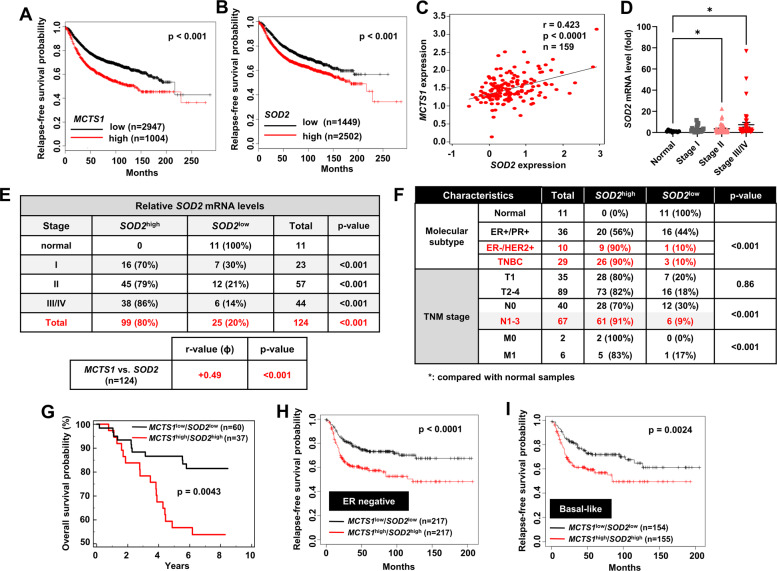
*SOD2*^high^/*MCTS1*^high^ expression is a new poor prognostic marker in invasive breast carcinoma. **A**, **B** The relapse-free survival (RFS) of breast cancer patients classified by low (*n* = 2947) and high (*n* = 1004) *MCTS1* expression (**A**) or by low (*n* = 1449) and high (*n* = 2502) *SOD2* expression (**B**) was evaluated using Kaplan–Meier (KM) Plotter analysis. **C** The correlation of *MCTS1* and *SOD2* gene expression in breast cancer patients (*n* = 159) was analyzed using the Pawitan dataset in the Oncomine cancer profiling database. **D–F** *SOD2* expression was analyzed in TissueScan cDNA arrays with normal breast tissue (*n* = 11) and breast tumor biopsies (*n* = 124) across distinctive tumor stages (**D**, **E**), molecular subtypes and TNM classifications (**F**). The *SOD2* mRNA levels in tumor samples were normalized to internal *ACTB* (β-actin) mRNA levels and then compared with the levels in normal breast samples. Data are presented as the mean ± s.e.m. **G** KM Plotter was used to estimate the survival of patients with *MCTS1*^high^/*SOD2*^high^ (*n* = 37) expression compared with that of those with *MCTS1*^low^/*SOD2*^low^ (*n* = 60) expression using the Pawitan dataset in the Oncomine database. **H**, **I** RFS was estimated for ER-negative (*n* = 434) (**H**) and basal-like (*n* = 309) (**I**) breast cancer patients with *MCTS1*^high^/*SOD2*^high^ or *MCTS1*^low^/*SOD2*^low^ expression using KM Plotter. Statistical analysis was performed using the log-rank Mantel–Cox test (**A**, **B**, and **G–I**), Pearson product-moment correlation coefficient (**C**), Kruskal–Wallis test followed by Dunn’s multiple comparison test (**D**) and *χ*^*2*^ test (**E**, **F**).

Using the Kaplan–Meier (KM) Plotter database [21] to inspect the clinical relevance of MCT-1 and MnSOD expression in breast cancer, we found that patients with high *MCTS1* (Fig. 1A) or high *SOD2* (Fig. 1B) expression showed a lower relapse-free survival (RFS) rate than those with low expression of *MCTS1* or *SOD2*. We previously identified that high MCT-1 expression is largely observed in breast cancer patients at both the initial and the late stages, and found in over than 70% of ER(+)/HER2(+) subtype and TNBC [20]. From the Oncomine cancer microarray database [22], we found that *MCTS1* and *SOD2* expression was positively correlated in breast cancer patients (*p* < 0.0001) (Fig. 1C). Further characterization of *SOD2* mRNA levels was conducted with breast cancer cDNA arrays (OriGene). *SOD2* expression was enriched among stage II (3.57-fold) and advanced-stage (III/IV) (7.36-fold) tumor tissues (Fig. 1D) relative to normal breast tissues. High *SOD2* expression was detected in 80% of breast cancer patients (Fig. 1E) and was positively correlated with *MCTS1* (*p* < 0.001). Strikingly, high *SOD2* expression was widely observed in aggressive HER2(+) subtype (90%) and TNBC (90%) tissues (Fig. 1F), while fewer in ER(+)/HER2(+) subtype (56%) and none of the normal breast tissues (0%) exhibited high *SOD2*. Moreover, high *SOD2* was linked with larger tumors (T2-4) (Fig. 1F), lymph node metastasis (N1-3), and distant metastasis (M1). Importantly, breast cancer patients with an *MCTS1*^high^/*SOD2*^high^ pattern demonstrated a lower overall survival rate than patients with an *MCTS1*^low^/*SOD2*^low^ pattern (*p* = 0.0043) (Fig. 1G). RFS probabilities in the ER-negative (*p* < 0.0001) (Fig. 1H) and basal-like (*p* < 0.0024) (Fig. 1I) subtypes of breast cancer were much higher in patients with an *MCTS1*^low^/*SOD2*^low^ pattern than in those with an *MCTS1*^high^/*SOD2*^high^ pattern. Collectively, the induction of MCT-1 and MnSOD is associated with breast cancer aggressiveness and patient with a poor prognosis.

### MCT-1 stabilizes Nrf2 to transcriptionally induce MnSOD

**Fig. 2:**
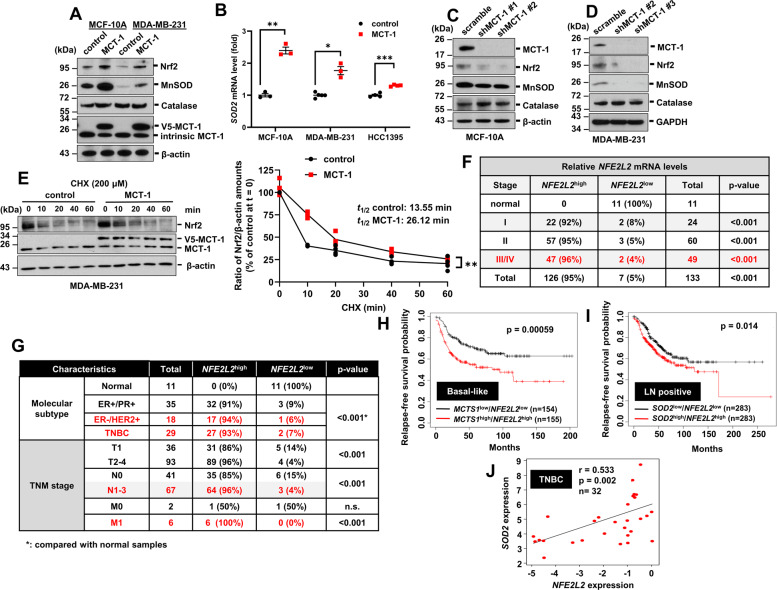
Overexpression of MCT-1 stabilizes Nrf2 and induces MnSOD. **A** MnSOD, Nrf2, and catalase levels were examined in normal MCF-10A cells and metastatic MDA-MB-231 cells. **B** The *SOD2* mRNA levels in MCF-10A, MDA-MB-231, and HCC1395 cells without (control) or with MCT-1 overexpression were measured by qRT-PCR. **C**, **D** MnSOD, Nrf2 and catalase levels were assayed upon MCT-1 gene silencing (shMCT-1 #1, #2 or #3) in MCF-10A (**C**) and MDA-MB-231 (**D**) cells. **E** Upon cycloheximide (CHX) treatment for the indicated times, Nrf2 protein stability and its amount relative to that of internal *ACTB* (right) were compared in MDA-MB-231 cells (control vs. MCT-1). Representative images (left) from four independent experiments are shown. **F**, **G** *NFE2L2* mRNA levels were analyzed across distinctive breast tumor stages (**F**) and molecular subtypes or TNM classifications (**G**) using TissueScan breast cancer cDNA arrays. The *NFE2L2* mRNA levels in tumor samples were normalized to the internal *ACTB* mRNA level and then compared with the levels in normal breast samples. **H-I** KM Plotter analysis estimated relapse-free survival in basal-like (**H**) breast cancer patients with *MCTS1*^high^/*NFE2L2*^high^ (*n* = 155) expression versus those with *MCTS1*^low^/*NFE2L2*^low^ (*n* = 154) expression and in breast cancer patients with lymph node metastasis (LN positive) (**I**) stratified by a *SOD2*^high^/*NFE2L2*^high^ (*n* = 283) or *SOD2*^low^/ *NFE2L2*^low^ (*n* = 283) signature. **J** The correlation of *SOD2* and *NFE2L2* expression in TNBC patients was analyzed using the Stickeler dataset (*n* = 32) in the Oncomine database. Data (**B**, **E**) are presented as the mean ± s.e.m. Statistical analysis was performed using a two-tailed unpaired Student’s *t*-test (**B**), linear regression followed by an analysis of variance (ANOVA) (**E**), the *χ*^2^ test (**F**, **G**), the log-rank Mantel–Cox test (**H**, **I**), and the Pearson product-moment correlation coefficient (**J**). *p < 0.05; **p < 0.01; ***p < 0.001.

To examine how MCT-1 promotes MnSOD, V5-tagged MCT-1 was introduced into nontumorigenic human mammary epithelial cells (MCF-10A) and the TNBC cell lines HCC1395 (derived from early stage TNBC) and MDA-MB-231 (IV2-3) (a highly invasive subline derived from two rounds of in vivo selection of lung metastases) [23]. We found that MCT-1 overexpression elevated the MnSOD protein level (Fig. 2A) and the level of nuclear factor-E2-related factor 2 (Nrf2 or *NFE2L2*), a transcriptional inducer of MnSOD. MCT-1 enrichment also led to increased *SOD2* mRNA in MCF-10A cells and the TNBC cell lines (Fig. 2B). Conversely, Nrf2 and MnSOD were reduced while MCT-1 silencing in MCF-10A (shMCT-1 #1 and #2) and MDA-MB-231 (IV2-3) (shMCT-1 #2 and #3) cells (Fig. 2C, D). However, overexpressing or silencing MCT-1 did not affect catalase (Fig. 2A, C, D), the H_2_O_2_ scavenger.

Constitutive Nrf2 activity occurs in cancer cells via either elevated Nrf2 transcription or disrupted Nrf2 turnover [24]. We found that *NFE2L2* mRNA were unchanged in both the MCF-10A and MDA-MB-231 (IV2-3) cell lines with or without MCT-1 overexpression (Supplementary Fig. S1A). However, upon inhibition of de novo protein synthesis with cycloheximide, MCT-1 overexpression extended the half-life (*t*_1/2_) of Nrf2 to approximately two times longer than that observed with control cells (Fig. 2E). Thus, MCT-1 upregulates Nrf2 by enhancing its stability, which in turn activates MnSOD transcription.

Consistent with the high *SOD2* identified in HER2(+) and TNBC samples (Fig. 1F), high *NFE2L2* expression was also identified in advanced stages (III/IV) (96%) (Fig. 2F), in the subtypes HER2(+) (94%) and TNBC (93%) and in patients with metastasis to the lymph nodes (N1-3) (96%) or the distant metastasis (M1) (100%) (Fig. 2G). Breast cancer patients with high *NFE2L2* expression had a lower RFS rate than those with low *NFE2L2* expression (*p* < 0.001) (Supplementary Fig. S1B). Patients with the basal-like subtype of invasive breast cancers and *MCTS1*^high^/*NFE2L2*^high^ expression exhibited a poorer prognosis than those with *MCTS1*^low^/*NFE2L2*^low^ expression (*p* = 0.00059) (Fig. 2H).

*MCTS1* and *NFE2L2* expression was strongly correlated (*p* < 0.0001) (Supplementary Fig. S1C). Breast cancer patients with lymph node metastasis (LN positive) who had *SOD2*^high^/*NFE2L2*^high^ expression displayed a relatively low RFS rate (*p* = 0.014) (Fig. 2I). TNBC patients exhibited a highly positive correlation between *SOD2* and *NFE2L2* expression (*p* = 0.002) (Fig. 2J). Overall, the upregulation of MnSOD and Nrf2 strongly correlates with breast cancer aggressiveness.

### MCT-1/MnSOD axis exacerbates TNBC cell malignant behaviors and curbs macrophage functions

**Fig. 3:**
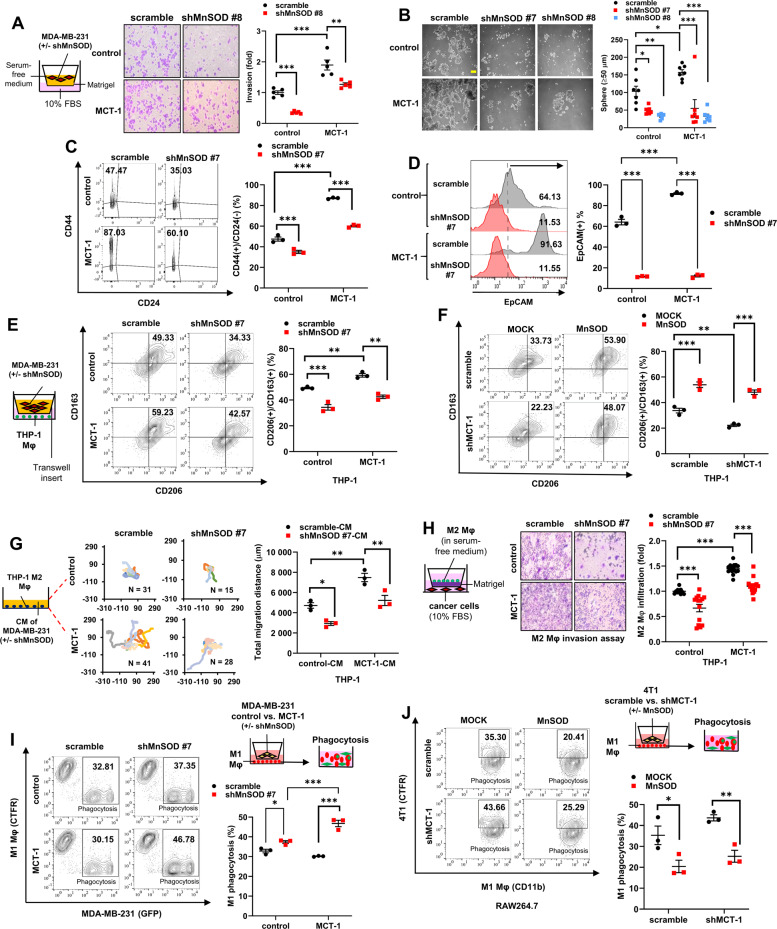
MCT-1/MnSOD signaling exacerbates breast cancer stemness phenotypes and M2 macrophage functions. **A** MDA-MB-231 cell invasion was assayed in a Transwell invasion chamber in serum-free conditions. Relative invasive ability was normalized to that of scrambled control cells. **B** MDA-MB-231 mammosphere formation (≥50 µm in diameter) was analyzed. Scale bar, 50 µm. **C**, **D** Flow cytometric analysis of CD44(+)/CD24(−) (**C**) and EpCAM(+) (**D**) populations in mammospheres (day 13). **E** M0 THP-1 macrophages were seeded in the lower chamber. MDA-MB-231 cells (control vs. MCT-1) with or without MnSOD depletion (scramble vs. shMnSOD) were seeded in the upper chamber of the Transwell system. CD163(+)/CD206(+) M2 macrophages were quantified (middle, right) after 48 h of coculture. **F** CD163(+)/CD206(+) M2 macrophages were measured after M0 macrophages were cocultured with MDA-MB-231 cells (scramble vs. shMCT-1) with or without MnSOD overexpression (MOCK vs. MnSOD) for 48 h. **G** The total migration distance of M2 THP-1 macrophages was measured in conditioned medium (CM) (right) of MDA-MB-231 cells with or without MnSOD knockdown (scramble vs. shMnSOD) and MCT-1 induction (control vs. MCT-1). Macrophage trajectories (left) emanating from the origin were plotted. **H** Invasion of M2 macrophages (right) was examined using a Boyden chamber Transwell system (left) as cocultured with MDA-MB-231 cells with or without MCT-1 induction (control vs. MCT-1) and MnSOD knockdown (scramble vs. shMnSOD). **I** M1 macrophages were exposed to MDA-MB-231 cells with or without MnSOD knockdown (scramble vs. shMnSOD) and MCT-1 induction (control vs. MCT-1) for 48 h. Phagocytosis of CellTrace Far Red (CTFR)(+) M1 macrophages against GFP(+) MDA-MB-231 parental cells was analyzed. **J** 4T1 cells (scramble vs. shMCT-1) with or without MnSOD induction (MOCK vs. MnSOD) were primed M1 RAW264.7 murine macrophages for 48 h. The phagocytic activity of CD11b(+) M1 RAW264.7 macrophages against CTFR(+) 4T1 parental cells was studied. Data are presented as the mean ± s.e.m. Statistical analysis was performed using two-way analysis of variance (ANOVA) followed by Tukey–Kramer *post hoc* analysis. *p < 0.05; **p < 0.01; ***p < 0.001.

To investigate whether MnSOD accounts for EMT progression, we generated MDA-MB-231 (IV2-3) cells with stable knockdown of MnSOD (shMnSOD) using a lentivirus-delivered short hairpin RNA construct (clones #7 and #8) (Supplementary Fig. S2A). In vitro wound-healing (Supplementary Fig. S2B) and invasion (Fig. 3A) assays confirmed that shMnSOD reduced cell migration and invasion regardless of the MCT-1 status. Matrix metalloproteinases (MMPs) control cancer cell dissemination and invasion [25]. As detected by in-gel zymography, ectopic MCT-1 expression promoted higher MMP-9 and MMP-2 activities than control cells (Supplementary Fig. S2C), and shMnSOD effectively decreased MMP-2 but only modestly reduced MMP-9 in cancer cells compared with the scramble control. Thus, MnSOD acts downstream of MCT-1.

Self-renewing subpopulation of breast cancer stem cells (BCSCs) facilitates cancer initiation, relapse, and metastatic colonization in primary and secondary sites [26]. Examining the potential of MnSOD in the cancer stemness of TNBC, we found that shMnSOD in MDA-MB-231 (IV2-3) cells suppressed the formation of BCSCs, demonstrating a reduced capacity for self-renewal (Fig. 3B), either with or without MCT-1 overexpression. Mammospheres with shMnSOD showed reduced *EPCAM* (Supplementary Fig. S2D), *NANOG*, *SOX2*, *SNAI1*, *PROM1*, and *ALDH1A1*. Compared with scrambled controls, shMnSOD mammospheres exhibited smaller CD44(+)/CD24(−) (Fig. 3C) and EpCAM(+) (Fig. 3D) subpopulations, revealing that shMnSOD diminished TNBC stemness even in the MCT-1 oncogenic background.

To confirm whether MnSOD induction can override the antioncogenic effects, MnSOD (Myc-DDK-tagged) was overexpressed in MDA-MB-231 (IV2-3) cells with MCT-1 knockdown (shMCT-1 #2) (Supplementary Fig. S2E). Indeed, the MnSOD overexpression restored the transforming ability inhibited by shMCT-1 (Supplementary Fig. S2F), thus profoundly promoting anchorage-independent growth. MnSOD also enriched the formation of mammospheres (Supplementary Fig. S2G) and the CD44(+)/CD24(-) subpopulations (Fig. Supplementary S2H), overpowering shMCT-1 effect.

To investigate whether shMnSOD in TNBC cells reprograms protumorigenic M2 macrophages, we established a Transwell coculture system with M0 macrophages and MDA-MB-231 (IV2-3) cells with or without MCT-1 overexpression and MnSOD knockdown. M0 macrophages were derived from monocytic THP-1 cells after stimulation with phorbol 12-myristate 13-acetate (PMA) (Supplementary Fig. S3A) and thus referred to as THP-1 macrophages, marked by increased pan-macrophage marker *ADGRE1* (F4/80) (Supplementary Fig. S3B, C, D). Upon coculture, shMnSOD cells inhibited THP-1 macrophage polarization into CD163(+)/CD206(+) M2 macrophages (Fig. 3E) with reductions in the *CD163* and *MRC1* (CD206) mRNA (Supplementary Fig. S3E) and intracellular M2 markers (Arginase-1 and IL-10) (Supplementary Fig. S3F), which were induced by coculture with the MCT-1-overexpressing cells. Inversely, MnSOD overexpression in MDA-MB-231 (IV2-3) cells with or without MCT-1 knockdown (shMCT-1) expanded the populations of CD163(+)/CD206(+) M2 macrophages (Fig. 3F). Hence, MnSOD activity in TNBC cells can modulate macrophage plasticity.

To analyze macrophage functions in response to TNBC cells, THP-1 macrophages were stimulated with lipopolysaccharides (LPS) to trigger M1 phenotypes or treated with IL-4 and IL-13 to induce M2 phenotypes, as previously described [27]. M2 macrophages showed higher mobility than M0 and M1 macrophages (Supplementary Fig. S3G). When we tested the chemotaxis in conditioned medium (CM) from MDA-MB-231 (IV2-3) cells, M2 macrophage mobility was promoted by CM from MCT-1-overexpressing cells (MCT-1-CM) compared with CM from control cells (control-CM) (Fig. 3G), but M2 mobility was substantially reduced in CM from shMnSOD cells. Similarly, in a Boyden chamber coculture system, MCT-1-overexpressing cells attracted M2 macrophages penetration (Fig. 3H), but shMnSOD cells suppressed the effect. Clearly, MCT-1/MnSOD in TNBC cells promotes M2 polarization, migration, and invasion.

M1 macrophages inhibit tumor progression and metastasis by phagocytosing cancer cells [4]. To determine whether TNBC cells can educate M1 macrophages that exhibit this distinctive phagocytic capacity, M1 THP-1 macrophages were labeled with CellTrace Far Red (CTFR(+)) and cocultured with green fluorescent protein (GFP)(+) MDA-MB-231 (IV2-3) cells. When MDA-MB-231 (IV2-3) cells were engulfed by macrophages, in vitro phagocytosis, i.e., CTFR(+)/GFP(+) macrophages, can be quantified by flow cytometry analysis (Supplementary Fig. S3H). M1 THP-1 macrophages had the highest phagocyticity against cancer cells among all macrophage types (Supplementary Fig. S3I). Consistently, LPS-induced murine CD11b(+) M1 RAW264.7 macrophages exhibited the strongest phagocytosis of CTFR(+) murine TNBC 4T1 cells (Supplementary Fig. S3J). Significantly, the phagocyticity of M1 THP-1 macrophages was improved when reacted with MDA-MB-231 (IV2-3) cells with shMnSOD (Fig. 3I). Nevertheless, M1 RAW264.7 macrophages showed reduced phagocyticity when faced with MnSOD-overexpressing 4T1 cells (Fig. 3J). Overall, MnSOD depletion inhibits TNBC cell malignancies, impairs M2 macrophage movements, and enhances M1 macrophage phagocytosis.

### MnSOD acts as a prooxidant that induces mROS generation and IL-6 secretion

**Fig. 4:**
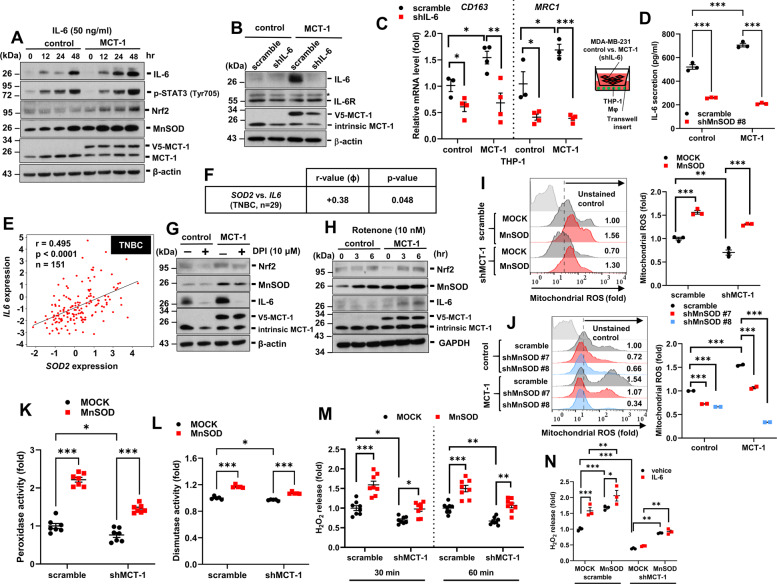
The MCT-1/IL-6/MnSOD axis induces mROS generation and M2 macrophage polarization. **A** The amounts of Nrf2 and MnSOD in MDA-MB-231 cells (control vs. MCT-1) were examined upon IL-6 (50 ng/ml) stimulation for different intervals. **B** IL-6 knockdown (shIL-6) was confirmed in MDA-MB-231 cells (control vs. MCT-1). **C** The expression of M2 markers (*CD163* and *MRC1*) were examined after THP-1 macrophages were cocultured with MDA-MB-231 cells (control vs. MCT-1) with or without shIL-6. **D** Secreted IL-6 levels were measured to evaluate MDA-MB-231 cells with or without MnSOD knockdown (scramble vs. shMnSOD) and MCT-1 overexpression (control vs. MCT-1). **E** The correlation of *SOD2* and *IL6* expression in TNBC patients (*n* = 151) was analyzed using the Gluck dataset in the Oncomine. **F** The correlation of *SOD2* and *IL6* expression in TNBC patients (*n* = 29) was examined using TissueScan breast cancer cDNA arrays. **G**, **H** Nrf2, MnSOD, and IL-6 levels were assayed after exposure to DPI (**G**) or Rotenone (Rot) (**H**). **I** Mitochondrial ROS (mROS) levels were quantified in MDA-MB-231 cells (scramble vs. shMCT-1) with or without MnSOD overexpression (MOCK vs. MnSOD) using superoxide fluorescent probe MitoSOX. **J** mROS levels were quantified in MDA-MB-231 cells (control vs. MCT-1) with or without MnSOD silencing (scramble vs. shMnSOD #7 and #8). **K**, **L** The peroxidase (**K**) and MnSOD dismutase activities (**L**) of MDA-MB-231 cells with or without MCT-1 knockdown (scramble vs. shMCT-1) and MnSOD overexpression (MOCK vs. MnSOD). **M** H_2_O_2_ release from MDA-MB-231 cells with or without MCT-1 knockdown (scramble vs. shMCT-1) and MnSOD overexpression (MOCK vs. MnSOD) was measured using Amplex Red. **N** Quantification of H_2_O_2_ released by MDA-MB-231 cells (scramble vs. shMCT-1) in MOCK vs. MnSOD expression conditions upon IL-6 stimulation for 24 h. Data are presented as the mean ± s.e.m. Statistical analysis was performed using two-way analysis of variance (ANOVA) followed by Tukey–Kramer (C, D, I–K, M-N) or Newman–Keuls (L) *post hoc* analysis or the Pearson correlation coefficient (**E**, **F**). *p < 0.05; **p < 0.01; ***p < 0.001.

Nrf2 transcriptionally activates IL-6 expression via an ARE on the IL-6 promoter in response to oxidative stress [14]. To examine whether IL-6 has an autocrine effect on Nrf2/MnSOD, MDA-MB-231 (IV2-3) cells were exposed to IL-6 that activated Tyrosine phosphorylation (Y705) of STAT3 and also enriched Nrf2 and MnSOD in a time-dependent manner (Fig. 4A). IL-6 stimulation also enhanced the mROS level (Supplementary Fig. S4A), particularly in MCT-1-overexpressing cells.

To validate whether IL-6 is responsible for the promotion of M2 polarization, IL-6 was knocked down in MDA-MB-231 (IV2-3) cells by introducing a lentivirus-delivered short hairpin RNA construct. This construct silenced IL-6 (shIL-6) and reduced the MCT-1 level (Fig. 4B). IL-6-deficient TNBC cells failed to induce THP-1 cell polarization into M2 macrophages (Fig. 4C), as indicated by decreases in the *CD163* and *MRC1*. Intriguingly, shMnSOD diminished IL-6 secretion induced by MCT-1-overexpressing MDA-MB-231 (IV2-3) cells (Fig. 4D). Clinically, TNBC patients revealed that *SOD2* strongly correlated with *IL6* expression, both in the Gluck dataset (*p* < 0.0001) (Fig. 4E) and in the TNBC cDNA arrays (OriGene) (*p* < 0.048) (Fig. 4F). Therefore, high MCT-1 and MnSOD in TNBC cells can induce IL-6 excretion that acts on macrophages to promote M2 polarization. IL-6 induces Nrf2 and MnSOD, thus providing a positive feedback to lock the cells in an oncogenic state.

Complex I is the key enzyme in the mitochondrial electron transport chain and is the major source of mROS [28]. To investigate the nexus between mROS and the IL-6/MnSOD circuit, MDA-MB-231 (IV2-3) cells were treated with diphenyleneiodonoium (DPI), an inhibitor of complex I that targets the flavin prosthetic group region [28], to block reverse electron transport-ROS formation. DPI reduced mROS production (Supplementary Fig. S4B) and decreased Nrf2 (Fig. 4G), MnSOD and IL-6 promoted by MCT-1. However, MDA-MB-231 (IV2-3) cells treated with a different inhibitor of complex I that targets the iron–sulfur cluster N2 [28], Rotenone (Rot), enhanced mROS production during forward electron transfer (Supplementary Fig. S4C) and consequently induced Nrf2, MnSOD and IL-6 in a time-dependent fashion (Fig. 4H), especially in MCT-1-overexpressing cells. Thus, the enhanced mROS/IL-6/MnSOD signaling loop is existed in TNBC cells.

Under pathophysiological conditions, dysregulated MnSOD expression fails to provide antioxidant protection (ROS dismutation, tumor suppression), converting the activity of MnSOD into prooxidant peroxidase activity (ROS induction, procarcinogenic) that harnesses H_2_O_2_ to activate a downstream signaling [29–32]. As we examined the crosstalk between MCT-1 and MnSOD related to mROS production in MDA-MB-231 (IV2-3) cells, shMCT-1 indeed reduced mROS (Fig. 4I), but overexpressing MnSOD reversed the antioxidative effect of shMCT-1. Conversely, shMnSOD reduced the mROS promoted by MCT-1 overexpression (Fig. 4J). This directed us to speculate that MCT-1 induces MnSOD to gain a noncanonical function as a prooxidant. Notably, overexpressing MnSOD predominantly enhanced peroxidase activity in TNBC cells with more MCT-1 (scramble) than shMCT-1 (Fig. 4K). MnSOD overexpression also induced the superoxide dismutase activity of MnSOD in both scramble and shMCT-1 cells (Fig. 4L). However, MnSOD overexpression still yielded persistent H_2_O_2_ production when shMCT-1 diminished H_2_O_2_ production (Fig. 4M). Furthermore, IL-6 stimulation enhanced the promotive effect of MnSOD on H_2_O_2_ in cells with high MCT-1 (scramble) than with low MCT-1 (shMCT-1) (Fig. 4N). Together, IL-6 induces MCT-1, MnSOD, and mROS in TNBC cells. In a positive feedback loop, high MnSOD and mROS enhance IL-6 secretion. Under the oncogenic stress, MnSOD becomes a prooxidant rather than performing the expected antioxidant function.

### MnSOD promotes TNBC stemness and M2 macrophage invasiveness but suppresses M1 macrophage phagocyticity

**Fig. 5:**
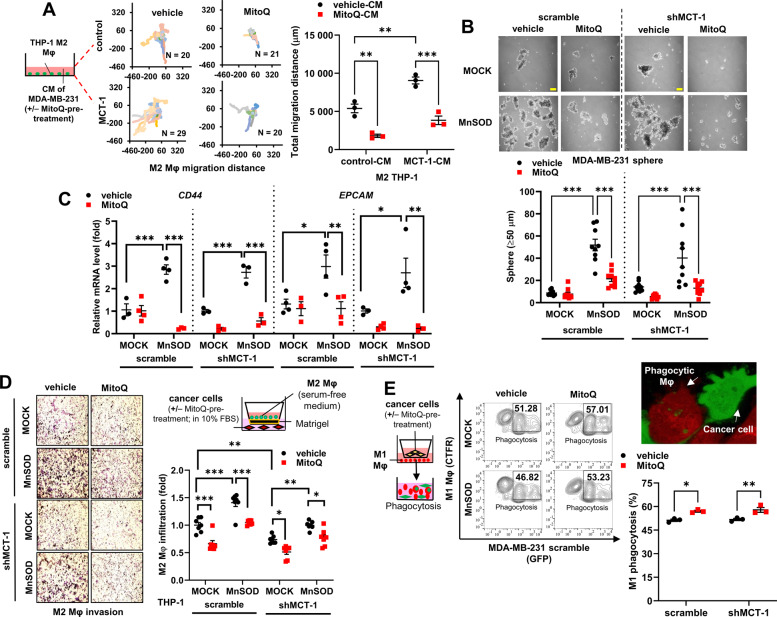
MitoQ inhibits TNBC stemness, M2 macrophage invasion, and M1 macrophage phagocyticity. **A** Quantification of the total migration distance (right) of M2 THP-1 macrophages after culture in CM from MDA-MB-231 cells (control, MCT-1) with or without MitoQ (mROS quencher) pretreatment (right). Macrophage trajectories (left) emanating from the origin were plotted using the DiPer program. **B** Without (vehicle) or with MitoQ treatment, mammosphere formation (≥50 µm in diameter) by MDA-MB-231 cells (scramble vs. shMCT-1) with or without MnSOD induction (MOCK vs. MnSOD) was quantified. Scale bar, 50 µm. **C** *CD44* and *EPCAM* mRNA levels were examined in MDA-MB-231 mammospheres (day 14) (scramble vs. shMCT-1) with or without MnSOD overexpression (MOCK vs. MnSOD) and without or with MitoQ treatment. **D** Invasion of M2 macrophages was assayed using a Boyden chamber system in response to vehicle- or MitoQ-pretreated MDA-MB-231 cells with or without MnSOD overexpression (MOCK vs. MnSOD). **E** MDA-MB-231 cells with or without MnSOD overexpression (MOCK vs. MnSOD) were pretreated with MitoQ or vehicle. CellTrace Far Red (CTFR)(+) M1 macrophages were primed with the pretreated cancer cells. M1 macrophage phagocytosis of parental GFP(+) MDA-MB-231 cells was analyzed by flow cytometry. Data are presented as the mean ± s.e.m. Statistical analysis was performed using two-way analysis of variance (ANOVA) followed by Tukey–Kramer *post hoc* analysis. *p < 0.05; **p < 0.01; ***p < 0.001.

Upregulated MnSOD exacerbates mROS generation and has been reported to promote cancer cell survival, metabolism, and metastasis [12, 32, 33]. To assess whether mROS drive the tumorigenic potential of MCT-1, MDA-MB-231 (IV2-3) cells (Supplementary Fig. S5A, B) and HCC1395 cells (Supplementary Fig. S5C, D) were exposed to Rotenone (Rot) (mROS inducer) and/or MitoQ (mROS inhibitor). Rot further enhanced the mROS (Supplementary Fig. S5A, C) and cancer cell invasiveness (Supplementary Fig. S5B, D) promoted by MCT-1, but adding MitoQ attenuated these effects. Although M2 macrophages exhibited higher mobility after priming with CM from MCT-1-overexpressing MDA-MB-231 (IV2-3) cells (Fig. 5A), the MitoQ-treated cancer cells inhibited the mobility of M2 macrophages. MitoQ inhibited the formation of mammospheres promoted by MnSOD overexpression (Fig. 5B), mostly in the shMCT-1 background. Interestingly, MnSOD-overexpressing MDA-MB-231 (IV2-3) cells were more sensitive to MitoQ than MOCK cells (Fig. 5C), as evident by the dramatic decline of BCSC markers such as *CD44* and *EPCAM*. Thus, MitoQ effectively inhibits TNBC cell mobility, stemness, especially in the shMCT-1 milieu.

In contrast, MnSOD overexpression in MDA-MB-231 (IV2-3) cells increased the invasiveness of M2 macrophages and counteracted the inhibitory effect of MitoQ on M2 invasion in a Boyden chamber system (Fig. 5D). Combined effect of MitoQ and shMCT-1 better suppressed M2 invasion. CTFR(+) M1 macrophage priming with the MitoQ-treated MDA-MB-231 cells modestly increased their phagocytic capabilities against GFP(+) MDA-MB-231 cells (Fig. 5E). Hence, overexpressing MnSOD in TNBC cells exhibits prooxidant activity that enhances mROS, cancer stemness, and M2 macrophage invasiveness.

### MnSOD deficiency inhibits TNBC progression and M2 macrophage infiltration

**Fig. 6:**
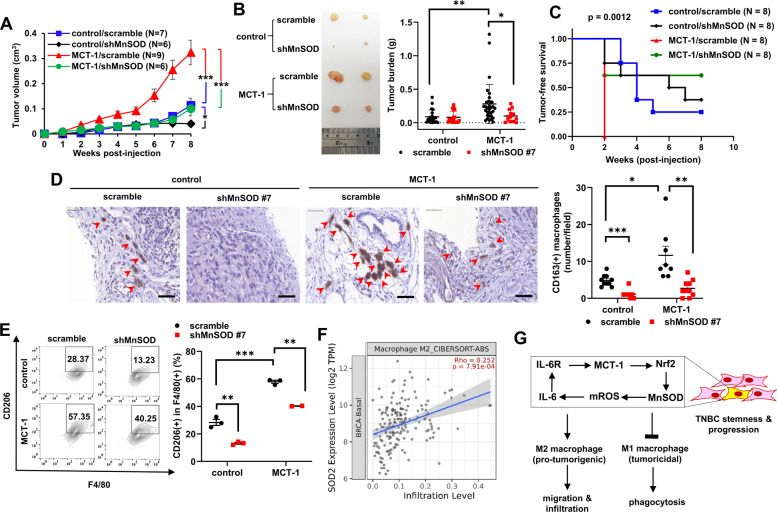
Targeting MnSOD suppresses TNBC progression and M2 macrophage infiltration. **A** MDA-MB-231 cells (1 × 10^6^) with or without MCT-1 overexpression (control vs. MCT-1) and MnSOD knockdown (scramble vs. shMnSOD) were orthotopically implanted into the mammary fat pad of nude mice (*n* = 6 ~ 9). Tumor growth rates were monitored weekly. **B** Representative tumor images (left) and tumor burdens (righ) were assessed at week 9 in xenograft mice. **C** Tumor mortality was analyzed after orthotopic injection of the indicated MDA-MB-231 cells into nude mice (*n* = 8). **D** Immunohistochemistry was used to examine tumor-associated CD163(+) M2 macrophages (denoted by red arrowheads). Scale bar, 50 µm. **E** Flow cytometric analysis of M2 macrophages (CD206(+) in F4/80(+) populations) residing in tumors was performed. **F** The correlation of the *SOD2* expression in invasive breast carcinoma patients of basal-like subtypes (*n* = 191) in The Cancer Genome Atlas (TCGA) cohort with the infiltration of M2 macrophages was evaluated using TIMER 2.0 and analyzed by the CIBERSORT-ABS tool. Expression levels are indicated as log2 transcripts per million (TPM). **G** Schematic figure depicting that amplification of the IL-6/MCT-1/Nrf2/MnSOD signaling loop aggravates TNBC progression/stemness and enhances M2 functions. MnSOD silencing thereby abolishes TNBC growth and improves M1 macrophage-mediated phagocytosis. Data are presented as the mean ± s.e.m. Statistical analysis was performed using a two-tailed unpaired Student’s *t*-test (**A**, **D**), the log-rank Mantel–Cox test (**C**), two-way analysis of variance (ANOVA) followed by Tukey–Kramer *post hoc* analysis (**B**, **E**), and the Spearman’s rank correlation coefficient (Rho) (**F**). *p < 0.05; **p < 0.01; ***p < 0.001.

To examine whether shMnSOD restricts TNBC progression, MDA-MB-231 (IV2-3) cells were engrafted into the mammary fat pads of BALB/c athymic nude mice. We identified that shMnSOD reduced tumor growth (Fig. 6A) and the tumor mass (Fig. 6B), even under the MCT-1 oncogenic effect. Orthotopic xenograft mice indicated that MCT-1-overexpressing cells were more tumorigenic than control cells (Fig. 6C), but shMnSOD mice showed dramatically reduced tumor mortality compared with mice harboring MCT-1-overexpressing cells (*p* = 0.0012). By immunohistochemistry analysis (Supplementary Fig. S6), MCT-1-overexpressing tumors without MnSOD silencing (MCT-1/scramble) exhibited more intratumoral CD163(+) M2 macrophage accumulation compared with control/scramble tumors (Fig. 6D, indicated by arrowheads), but M2 macrophage numbers were profoundly reduced in both control tumors and MCT-1 tumors with shMnSOD relative to scramble cohorts. The populations of tumor-associated CD206(+) of F4/80(+) M2 macrophages were larger in the MCT-1 tumors than in the control tumors (Fig. 6E), but they were significantly decreased by shMnSOD in both groups. Similarly, *SOD2* expression was positively correlated with M2 macrophage infiltration in patients with basal-like aggressive breast carcinoma (*p* < 0.001) (Fig. 6F), as validated by TIMER 2.0 [34], a systematic analysis of tumor-infiltrating immune cells. Collectively, MnSOD deficiency diminishes TNBC growth, mortality, and immunosuppressive M2 macrophages (Fig. 6G). Blocking the MCT-1/IL-6/Nrf2/MnSOD axis and excessive mROS with antioxidants such as DPI and MitoQ are potential therapeutics that can reshape TME and inhibit TNBC aggressiveness.

## Discussion

MnSOD is typically known as a tumor suppressor due to its antioxidant role in protecting cells from oxidative damage [9]. However, the roles of MnSOD in TME modulation have been understudied. We now show that the oncogene MCT-1 induces Nrf2/MnSOD and mROS. MnSOD impacts TNBC cell aggressiveness, but MnSOD deficiency suppresses M2 macrophage phenotypes, the TNBC cell malignancies, TNBC progression, and M2 infiltration.

Prior investigation has shown that inducing oncogenic v-Src kinase in MCF-10A cells potentiates MnSOD expression [11, 12]. High MnSOD expression in MCF-7 luminal breast cancer cells increases H_2_O_2_ [11], which sustains the Warburg effect. Using a multistage skin carcinogenesis mouse model with human MnSOD promoter-enhancer elements [10], MnSOD transcripts upregulated at the advanced stage due to p53 loss. This also matches our findings that oncogenic MCT-1 increased MnSOD in p53-mutant aggressive MDA-MB-231 (IV2-3) cells, and MCT-1 reduces wild-type p53 in MCF-10A cells and invasive A549 cancer cells [19], leading to MnSOD induction. Besides, p53 binding sequences in the MnSOD promoter determine MnSOD expression in fibroblasts [35].

We found that MCT-1 overexpression drives the prooxidant function of MnSOD, in turn prompting TNBC cells to fight against and adapt to the oxidative environment. Similarly, increased acetylation of MnSOD on the lysine 68 (K68) residue can enhance prooxidant MnSOD activity [32], thereby exacerbating stemness reprogramming [12]. This current study concomitantly identifies that the prooxidant peroxidase MnSOD expands BCSCs and invasiveness in high MCT-1 conditions by augmenting mROS, and these malignant phenotypes can be reversed by quenching mROS with MitoQ. Diminishing mROS or silencing MnSOD prooxidant effect could be a novel therapeutic regimen for aggressive breast cancer.

We uncovered that MnSOD induction in the oncogenic MCT-1 occurs via Nrf2 stabilization. Comparably, under oncogenic K-Ras stimulation, increased transcription or protein stability of Nrf2 enhances pancreatic and mammary cancer progression in an mROS-dependent or -independent manner [33, 36]. Although Nrf2 binds to the ARE in the promoter region of multiple genes that control ROS detoxification [24], we found that MCT-1 overexpression does not alter catalase in TNBC cells. Likewise, increased MnSOD without catalase increment results in induced oxidative stress in fibroblasts and lymphoblasts [35].

Our study shows that IL-6/Nrf2/MnSOD positive feedback loop exists in TNBC cells, provoking mROS or H_2_O_2_ generation in TNBC cells. Similarly, IL-6 upregulates MnSOD in multiple myeloma and prostate cancer cells [37, 38], and Nrf2 mediates hepatic IL-6 induction in vivo [14]. Hence, despite inducing the IL-6/Nrf2 axis to upregulate MnSOD, the oncogenic MCT-1 activity enriched mROS by augmenting the Nrf2/MnSOD/IL-6 circuit. We first demonstrate that the MCT-1/IL-6/Nrf2/MnSOD/mROS axis is a molecular profile that instigates aggressiveness and stemness in TNBC, suggesting that this axis could be a favorable therapeutic target.

Importantly, we demonstrated for the first time that the M2 macrophage migration and invasion can be diminished by shMnSOD, MitoQ, or shMCT-1. In chemically induced colitis, mROS production in colon tissues activates the NF-κB pathway [39], leading to the enhanced recruitment of M2 macrophages and regulatory T cells. IL-4-induced M2 macrophages are susceptible to MnTE-2-Pyp^5+^, a redox-active drug that mimics the activity of superoxide dismutases [40]. Thus, mROS inhibitor could boost the macrophage-based anti-tumor immunity.

Our study also first indicated that shMnSOD and shMCT-1 enhanced the phagocytosis of TNBC cells by M1 macrophages, signifying that phagocytosis can be controlled by redox homeostasis and oncogenic signaling. Consistently, M1 macrophage phagocytosis was increased in the MitoQ-treated TNBC cells, indicating that depleted mROS facilitate M1 macrophage tumoricidal activity. H_2_O_2_ can travel from cell to cell and suppress the activation of bone marrow-derived macrophages [41]. Alternatively, H_2_O_2_ induces TNF-α production in macrophages and triggers M1 polarization by activating the p38/JNK pathway [42]. Hence, balancing mROS and restricting mROS outflow in aggressive cancer cells could help immune surveillance and modify the TME.

In conclusion, our results provide new insights into the mechanism by which the oncogene MCT-1 deregulates redox homeostasis by changing the antioxidant MnSOD to a prooxidant that produces excess IL-6 and mROS and promotes an immunosuppressive TME. Targeting MnSOD or mROS with redox-active drugs could facilitate the engulfment of cancer cells by M1 macrophages and inhibit M2 macrophage functions, EMT progression, cancer stemness, and tumor advancement.

## Materials and methods

### Cell culture and transfection

MCF-10A cells were cultured routinely in DMEM/F-12 (#12400024, Life Technologies, Grand Island, NY, USA) supplemented with 5% horse serum (#16050122, Life Technologies), 20 ng/ml recombinant human epidermal growth factor (#CYT-217, ProSpec, Ness Ziona, Israel), 0.5 mg/ml hydrocortisone (#3867, Calbiochem, San Diego, CA, USA), 100 ng/ml cholera toxin (#C8052, Sigma-Aldrich, St. Louis, MO, USA), 10 μg/ml insulin (#I0516, Sigma-Aldrich) and 100 U/ml penicillin/streptomycin (#SV30010, HyClone, Logan, UT, USA). MDA-MB-231 (IV2-3) parental cell line was a gift from Lu-Hai Wang (China Medical University, Taichung, Taiwan). MDA-MB-231 (IV2-3), HCC1395, 4T1, THP-1, and RAW264.7 cells were maintained in RPMI-1640 medium (#31800022, Life Technologies) supplemented with 10% fetal bovine serum (FBS) (#10437028, Life Technologies), 2 mM L-glutamine (#SH30034.01, HyClone) and 100 U/ml penicillin/streptomycin (#SV30010, HyClone). V5-tagged MCT-1 was stably transfected into MCF-10A, MDA-MB-231 (IV2-3), and HCC1395 cells as previously described [20], and the transfectants were maintained in complete medium containing 100 or 200 μg/ml neomycin (G418) (#345810, Millipore, Darmstadt, Germany). The MCT-1 gene was stably knocked down in MCF-10A, 4T1, and MDA-MB-231 (IV2-3) cells as previously described [20], and the transfectants were maintained in complete medium containing 0.5 or 10 μg/ml puromycin (#A1113802, Millipore) or 8 μg/ml blasticidin (#ant-bl, InvivoGen, San Diego, CA, USA). MnSOD knockdown was established using a lentiviral-based shRNA vector (pGFP-C-shLenti) (#TR30021, OriGene, Rockville, MD, USA). To generate stable transfectants for MnSOD knockdown, pGFP-C-shLenti carrying scrambled shRNA or shMnSOD was transfected into HEK293T cells with Lipofectamine 3000 (#L3000001, Thermo Fisher Scientific, Waltham, MA, USA). After transfection for 24 h, the medium was replaced with BSA-enriched medium, and then the conditioned medium was collected at 24 and 48 h. The lentivirus-containing medium was centrifuged to remove cell debris and used to transduce MDA-MB-231 (IV2-3) cells with polybrene transfection reagent (Millipore); then, the cells were selected with 20 μg/ml puromycin for 2 weeks. To generate cells overexpressing MnSOD, True-ORF pCMV6-Entry-Flag-tagged SOD2 (#RC202330) and Sod2 (#MR202568) (all from OriGene) were transfected into MDA-MB-231 (IV2-3) and 4T1 cells with Lipofectamine 3000 (Thermo Fisher Scientific), and the transfectants were selected with 100 or 200 μg/ml neomycin (G418) for 2 weeks. IL-6 knockdown in MDA-MB-231 (IV2-3) cells was established as previously described [20], and the cells were maintained in complete medium containing 0.5 μg/ml puromycin. Cell lines were not authenticated using STR profiling, but all cell lines were kept at low passages in order to maintain their identity. Generated stable cell lines were authenticated via Western blots to validate high and/or low expression of MCT-1/MnSOD and routine observation of cell morphology under the microscope.

### Western blot analysis and antibodies (Abs)

Western blotting was conducted as previously described [43]. Abs against MnSOD and FLAG-tag were from Enzo Life Sciences (#ADI-SOD-111, Farmingdale, NY, USA) and Sigma-Aldrich, respectively; Abs against Nrf2 (#12721), IL-6 (#12153), phospho-STAT3 (Tyr705) (#9145), Catalase (#14097), Arginase-1 (#93668) and IL-10 (#12163) were from Cell Signaling Technology (Danvers, MA, USA); Abs against endogenous MCT-1 (#H00028985-M01A) were from Abcam (Cambridge, UK); and Abs against IL-6R (#GTX54364), V5-MCT-1 (#GTX117793), F4/80 (#GTX26640), β-actin (#GTX109639), and GAPDH (#GTX100118) were from GeneTex (Irvine, CA, USA).

### Cycloheximide chase assay

Cells (8 × 10^5^) were seeded in a 60 mm dish with complete medium and incubated for 24 h. The medium was then removed and replaced with a medium containing 200 µM cycloheximide (#C7698, Sigma-Aldrich). Cell lysates were collected at the indicated time intervals and subjected to Western blotting.

### Mitochondrial ROS level

mROS levels were quantified with MitoSOX Red (#M36008, Molecular Probes, Eugene, OR, USA) using a FACSCalibur flow cytometer (BD Biosciences, San Jose, CA, USA) as previously described [19] and analyzed using FlowJo v10 (BD Biosciences). For experiments involving an mROS inducer or quencher, cells were treated with or without 50 ng/ml IL-6 (#200-06, PeproTech, Rocky Hill, NJ, USA), 10 nM Rotenone (Sigma-Aldrich) or 0.5 μM MitoQ (#89950, Cayman Chemical, Ann Arbor, MI, USA) before MitoSOX loading.

### H_2_O_2_ release and peroxidase activity

H_2_O_2_ released by the cells and peroxidase activity were measured by Amplex Red reagent (#A22188, Invitrogen, Carlsbad, CA, USA) according to the manufacturer’s instructions. The absorbance at 560 nm was measured using a Tecan Infinite 200 PRO multimode microplate reader (Tecan, Männedorf, Switzerland). For the H_2_O_2_ assay involving IL-6 induction, cells were pretreated with or without 50 ng/ml IL-6 (#200-06, PeproTech) before incubation with Amplex Red.

### MnSOD dismutase activity

Superoxide dismutase activity in the sonicated cell homogenates was detected using the Superoxide Dismutase Assay Kit (#706002, Cayman Chemical) according to the manufacturer’s protocol. MnSOD activity was measured after incubating the reaction mixture with 2 mM potassium cyanide (KCN) (gift from Institute of Biotechnology and Pharmaceutical Research at National Health Research Institute, Zhunan, Taiwan) for 30 min to inactivate CuZnSOD (SOD1) and extracellular SOD (SOD3) activity. The absorbance at 450 nm was monitored using a Tecan Infinite 200 PRO multimode microplate reader (Tecan).

### Measurement of IL-6 secretion

To analyze IL-6 secretion, cancer cells (8 × 10^5^) were seeded in a 60-mm culture dish with complete medium and incubated for 72 h. The complete medium was removed and replaced with 2 ml serum-free medium, and the cells were incubated for 48 h. The medium was collected by centrifugation at 2,000 rpm and 4 °C for 10 min. The supernatants were immediately used for analysis of IL-6 secretion by the Human IL-6 ELISA MAX^TM^ Deluxe Set (#430505, BioLegend, San Diego, CA, USA) according to the manufacturer’s protocol. Reactions were stopped with 2 N H_2_SO_4_, and the absorbance was read at 450 nm and 570 nm using a Tecan Infinite 200 PRO multimode microplate reader (Tecan). The absorbance of samples at 570 nm was subtracted from the absorbance at 450 nm.

### Conditioned medium production

To obtain MitoQ-treated conditioned medium (CM), MDA-MB-231 (IV2-3) cells (2 × 10^6^) were seeded in a 100 mm dish, cultured until 80% confluent, treated with or without 0.5 μM MitoQ (Cayman Chemical) in complete RPMI medium for 24 h, rinsed twice with phosphate-buffered saline (PBS) to remove MitoQ, replaced and cultured with medium containing 1% FBS for another 24 h. The CM was harvested, followed by centrifugation at 200 × g for 10 min and filtration through 0.22-µm-pore filters to remove cell debris, and directly used for the experiment or stored at −80 °C.

### Macrophage differentiation and migration

Humane THP-1 monocytes and M0 murine RAW264.7 macrophages were differentiated into M1 and M2 macrophages as previously described [27]. Migratory abilities of macrophages were analyzed under a Leica AF6000LX microscope (Leica Microsystems, Wetzlar, Germany) using a 20x objective. Images were acquired for 18–24 h at 5 min intervals. Cell movements were tracked with Metamorph (Molecular Devices, San Jose, CA, USA) to quantify the total migration distance. Cell movement tracks emanating from the initial position were plotted using the DiPer macros [44]. For macrophage migration in response to MitoQ (Cayman Chemical), M2 THP-1 macrophages were cultured with CM and the movement was monitored as described above.

### Macrophage polarity and invasion

MDA-MB-231 (IV2-3) (2 × 10^5^) cells were plated on 0.4-μm-pore cell culture inserts (#353090, Corning, Corning, NY, USA), and M0 THP-1 macrophages (1 × 10^6^) were seeded in the lower chamber of the 6-well plate, and then these cells were cocultured for 48 h. The polarized macrophages were harvested, resuspended, and immediately used for flow cytometry analysis. Macrophage invasion toward cancer cells was assayed using Corning BioCoat Tumor Cell Invasion Systems (#354480, Corning, Bedford, MA, USA) as previously described [27]. Images were acquired using a Leica DM IRB (Leica Microsystems) with a 10x objective and analyzed using ImageJ (National Institutes of Health, Bethesda, MD, USA).

### Flow cytometry analysis

Macrophage polarity was characterized using the following Abs at 1:50 dilutions: Alexa Fluor 647-conjugated anti-CD163 (#562669, BD Biosciences) and BB515-conjugated anti-CD206 (#564668, BD Biosciences). In pan-macrophage marker analysis of THP-1, anti-F4/80 (Genetex) was used at 1:50, then followed by conjugation with Alexa Fluor 546 (#A-11010, Molecular Probes) at 1:100 dilution. For cancer stemness analysis, mammospheres were stained with the following Abs: BB515-conjugated anti-CD44 (#564582, BD Biosciences) and Alexa Fluor 647-conjugated anti-CD24 (#561644, BD Biosciences) at a 1:50 dilution or BB515-conjugated anti-EpCAM/CD326 (#565398, BD Biosciences) at a 1:10 dilution. BB515- (#564416) and Alexa Fluor 647-conjugated (#557714) mouse IgG1 κ isotype controls (BD Biosciences) were used as negative controls. To analyze TAMs, single cells isolated from breast tumor tissues (2 × 10^5^) were incubated with anti-CD16/CD32 (1:2000) (#553142, BD Biosciences) for 15 min at 4 °C to block nonspecific binding, stained with BB700-conjugated anti-F4/80 (#746070, BD Biosciences) and Alexa Fluor 647-conjugated anti-CD206 (#141712, BD Biosciences) antibodies at a 1:50 dilution. BB700- (#566413) and Alexa Fluor 647-conjugated (#400526) rat IgG2a κ isotype controls (BD Biosciences) were used as negative controls. In phagocytosis experiments with THP-1 M1 macrophages, phagocytic events were measured as the number of CTFR(+)/GFP(+) macrophages, which were quantified as a percentage of total CTFR(+) macrophages. Meanwhile, the phagocytosis of 4T1 cells by M1 RAW264.7 macrophages was indicated by the number of CTFR(+)/CD11b(+) cells compared with the total number of CD11b(+) macrophages. The BB515-conjugated anti-CD206, BB515-conjugated anti-CD44, BB515-conjugated anti-EpCAM, BB515-conjugated anti-CD11b antibodies were excited by a 488 nm laser line, and the emissions were detected in the FL1 channel (515-545 nm) with a 530/30 band-pass (BP) filter. Alexa Fluor 546-conjugated anti-F4/80 was excited by a 488-nm laser line, and the emissions were detected in the FL2 channel (564–606 nm) with a 585/42 BP filter. The BB700-conjugated anti-F4/80 antibody was excited by a 488 nm laser line, and the emission was detected in the FL3 channel with a 670 nm long-pass (LP) filter. The Alexa Fluor 647-conjugated anti-CD163, Alexa Fluor 647-conjugated anti-CD206, and Alexa Fluor 647-conjugated anti-CD24 antibodies were excited by a 635 nm laser line, and the emission was detected in the FL4 channel (653–669 nm) with a 661/16 BP filter. All flow cytometry experiments were performed on a FACSCalibur flow cytometer (BD Biosciences) and analyzed using FlowJo v10 (BD Biosciences).

### Flow cytometry-based phagocytosis assay

THP-1 monocytes were labeled with CellTrace Far Red (CTFR) (#C34564, Thermo Fisher Scientific) according to the manufacturer’s instructions and then polarized into M1 macrophages [27]. To provide activation and priming by cancer cells, M1 macrophages were cocultured with MDA-MB-231 (IV2-3) cells with MnSOD silencing (control vs MCT-1) or overexpression (scramble vs shMCT-1) for 48 h using Transwell inserts as previously described. The primed M1 macrophages were harvested and immediately tested for their ability to phagocytose cancer cells by directly incubating the M1 THP-1 macrophages (2 × 10^5^ cells) with MDA-MB-231 (IV2-3) cells (1 × 10^5^) carrying a GFP reporter for 2 h in a 37 °C incubator in serum-free RPMI medium (#31800022, Life Technologies) in ultralow-attachment 24-well plates (#3473, Corning, Kennebunk, ME, USA). For the phagocytosis assay involving mouse macrophages, murine 4T1 cell lines were prestained with CTFR and incubated with LPS-differentiated M1 RAW264.7 macrophages in serum-free RPMI medium for 2 h. All the cells were collected and stained with a BB515-conjugated anti-CD11b antibody (#564454, BD Biosciences) (1:50). Phagocytosis events were analyzed by flow cytometry.

### Cancer cell invasion and migration

Cell invasion assays were performed using Corning BioCoat Tumor Cell Invasion Systems (#354480, Corning) as previously described [19]. Cell migration was assayed using ibidi culture inserts (ibidi GmbH, Planegg, Germany). Cancer cells (3 × 10^4^) were seeded in a well containing a culture insert for 24 h. The culture inserts were then removed to create a cell-free gap. The remaining cells were immediately washed with warm PBS and overlaid with complete medium. Migratory cells that repaired the cell-free gap were monitored with a Leica AF6000LX microscope (Leica Microsystems) using a 10x objective lens. Live-cell images were acquired at three different positions per sample over 24 h at one-hour intervals. Wound closure capacity was analyzed with Metamorph (Molecular Devices).

### Mammosphere formation

Single-cell suspensions of MDA-MB-231 (IV2-3) cells with or without MnSOD silencing or overexpression were cultured in ultralow-attachment plates (Corning) at 4 × 10^4^ cells (6-well plate) or 1 × 10^4^ cells (24-well plate) in DMEM/F-12 (#12400024, Life Technologies) supplemented with 2 mM L-glutamine (#SH30034.01, HyClone), 100 U/ml penicillin/streptomycin (#SV30010, HyClone), 20 ng/ml recombinant human epidermal growth factor (#CYT-217, ProSpec), 20 ng/ml recombinant human basic fibroblast growth factor (#AF-100-18B, PeproTech), and 1x B27 supplement (#12587010, Invitrogen). The medium was replenished every 4 days for 14 ~ 16 days during culture in a 37 °C incubator. Mammosphere formation was imaged using a Nikon DIAPHOT300 inverted microscope (Nikon, Chiyoda-ku, Japan) at 200x magnification and quantified using ImageJ (National Institutes of Health), or harvested at the indicated times for flow cytometry.

### Soft agar colony formation assay

Anchorage-independent growth was characterized when MDA-MBA-231 cells (2 × 10^4^) were seeded on 0.3% agarose (#50002, Lonza, Rockland, ME, USA) in RPMI medium over a bottom layer of 0.6% agarose in RPMI medium. The cells were fed every 5 days with RPMI medium containing 0.3% agarose. After 2 weeks of incubation, the colonies were imaged with a Leica AF6000LX microscope using a 20x objective.

### Gelatin zymography study

MDA-MB-231 (IV2-3) cells (1 × 10^6^) were seeded in a 60 mm culture dish with complete RPMI medium and incubated for 24 h. The complete medium was removed, and the cells were refed with 0.5 ml RPMI containing 1% FBS. After 24 h of incubation, the medium was collected and centrifuged at 2,000 rpm and 4 °C for 10 min. The supernatants were resolved in an 8% SDS-PAGE gel containing 0.1% gelatin. Gels were subsequently washed in 2.5% (v/v) Triton X-100/PBS for 30 min and agitated in freshly prepared zymogram developing buffer (50 mM Tris-HCl, pH 7.5; 200 mM NaCl; 5 mM CaCl_2_; 0.02% Brij 35 detergent (#203724, EMD Chemicals, San Diego, CA, USA)) at 37 °C for 24 h. To visualize MMP activity, gels were stained with 0.5% (w/v) Coomassie Brilliant Blue R-250 (Sigma-Aldrich) for 30 min, immediately destained with the solution I (25% ethanol and 10% acetic acid) for 30 min, washed with solution II (5% ethanol and 7.5% acetic acid) for 4–5 h and agitated overnight in distilled water. Media containing 1% FBS or 10% FBS were used as negative and positive controls, respectively. Zymograms were scanned and analyzed using ImageJ (National Institutes of Health).

### Xenograft tumor growth and immunohistochemistry analysis

For examination of orthotopic mammary tumor growth, 6-week-old female BALB/c nude mice (cAnN.Cg-Foxn1^nu^/CrlNarl) (BioLASCO, Taipei, Taiwan) were randomly divided at the start of the experiment to be implanted with luciferase-expressing MDA-MB-231 (IV2-3) cells (1 × 10^6^), which were stably transfected with MnSOD-specific shRNA or scrambled shRNA, on the bilateral side of the 4th mammary fat pads. These cell lines were tested with EZ-PCR Mycoplasma Detection Kit and showed negative results for mycoplasma contamination (#20-700-20, Biological Industries, Kibbutz Beit-Haemek, Israel). Tumor development was measured weekly and recorded as tumor width (*W*) and tumor length (*L*). Tumor volume was calculated using the formula *V* = *1/2 (W*^*2*^ *×* *L)*. Observers were not blinded during tumor measurement because experimental identities were recorded on the cage cards. After implantation (9 weeks), the mice were euthanized by CO_2_ inhalation, and the tumors were collected for weight and size analysis, under a protocol approved by the NHRI Institutional Animal Care and Use Committee (NHRI-IACUC-107053-A). Immunohistochemistry was performed on MDA-MB-231 (IV2-3) tumor sections as previously described [20] with staining for CD163 (1:600) (#GTX35247, GeneTex).

### Tumor-associated macrophages

Tumor-associated macrophages were isolated from mammary MDA-MB-231 (IV2-3) tumors in xenografted mice according to an established protocol [27] with minor modifications. Tumors were minced and incubated with digestion buffer (1 mg/ml) collagenase D (#11088858001, Sigma-Aldrich), 0.25 mg/ml DNase I (#DN25, Sigma-Aldrich), and 0.25% (v/v) trypsin-EDTA solution (1X) (#SV30037.01, HyClone) in serum-free RPMI-1640 medium (Life Technologies) for 1 h at 37 °C under continuous agitation. The cell suspension was filtered using a 70 µm cell strainer, lysed with red blood cell lysis buffer (155 mM NH_4_Cl, 12 mM NaHCO_3_, and 0.1 mM EDTA), and fixed with 4% (v/v) paraformaldehyde for flow cytometry analysis.

### Quantitative RT-PCR (qRT-PCR)

To measure mRNA expression levels, cells were washed with ice-cold PBS and subjected to total RNA extraction with RNAzol RT (#RN190, Molecular Research Center, Cincinnati, OH, USA) according to the manufacturer’s protocol. Extracted RNA samples were immediately used for cDNA synthesis using the Maxima First Strand cDNA Synthesis Kit (#K1642, Thermo Fisher Scientific) according to the manufacturer’s instructions. cDNA samples (100 ng) were mixed with PrecisionPLUS qPCR Master Mix (#PPLUS, Primerdesign, Southampton, UK) and subjected to quantitative PCR using a ViiA 7 Real-time PCR System (Thermo Fisher Scientific). All qRT-PCR assays were analyzed using comparative Ct methods (2^−∆∆Ct^). Results were normalized to *ACTB* mRNA results as an internal control. ΔΔCT = [(Ct target gene − Ct internal control) of treated cells − (Ct target gene − Ct internal control) of untreated cells].

### Primer sequences

The primer sequences were designed according to the NCBI Probe Database and listed in Supplementary Table S1.

### Clinical study

The *SOD2* and *NFE2L2* mRNA levels in normal breast and breast tumor specimens were quantified by qRT-PCR using TissueScan Breast Cancer cDNA Arrays I (BRCT101), III (BRCT103), and IV (BRCT104) (OriGene). The results were normalized to those for *ACTB*, which was used as an internal control, and then compared to those for normal breast tissue. Data were analyzed using comparative Ct (cycle threshold) methods (2^−∆∆Ct^). ΔΔCT = Ct of breast tumor − Ct of a normal breast. The expression levels of the *MCTS1*, *SOD2*, and *NFE2L2* genes in breast cancer patients were also obtained from the Oncomine cancer profiling database (https://www.oncomine.org/resources/) [22]. Correlation plots between *MCTS1* and *SOD2*, *MCTS1* and *NFE2L2*, *SOD2* and *NFE2L2*, and *SOD2* and *IL6* derived from the Oncomine dataset were then generated and analyzed using Minitab 16 software (Minitab Ltd., Coventry, UK), while KM plots were generated and analyzed using MedCalc (MedCalc, Ostend, Belgium). Alternatively, the probability of relapse-free survival in breast cancer patients was also analyzed using KM Plotter (https://kmplot.com/analysis/) [21]. The cohorts were divided into high and low expression groups according to the upper/lower quartile or median expression of the gene of interest. Additionally, the correlation between *SOD2* expression and M2 macrophage infiltration in breast cancer patients in The Cancer Genome Atlas (TCGA) cohort was analyzed using TIMER 2.0. (http://timer.comp-genomics.org) [34].

### Statistics

Sample sizes was determined based on experience and preliminary study. For in vitro experiments, a minimum number of three biological replicates were used and shown in all figures. In vivo sample sizes were based on the previous studies in the laboratory that revealed that the number of animals to be sufficient to obtain significant differences. Cells were allocated into control and experimental groups based on its genetic manipulation (*MCTS1*, *SOD2*, or others). Investigators were not blinded during data collection and/or analysis for each in vitro experiment to allow correct identification of samples (control and other groups). Experiments were repeated at least twice on two independently grown cell cultures. The survival probability of breast cancer patients and tumor-free survival in mice were analyzed using the log-rank Mantel–Cox test. Correlations between *MCTS1* and *SOD2*, *MCTS1* and *NFE2L2*, *SOD2* and *NFE2L2*, and *SOD2* and *IL6* were examined by Pearson product-moment correlation coefficients, whereas the correlation between MnSOD expression and M2 macrophage infiltration was evaluated using Spearman’s rank correlation coefficient. The statistical significance of *SOD2* and *NFE2L2* mRNA in TissueScan Human Breast Cancer cDNA Arrays I, III, and IV (OriGene) was assessed by the chi-squared (*χ*^2^) test. Experiments involving qRT-PCR, cancer migration and/or invasion, cycloheximide chase, mROS quantification, ELISA, the enzymatic activity, mammosphere formation, macrophage polarization/migration/invasion/phagocytosis, H_2_O_2_ release, tumor burden, and tumor growth were analyzed with one-way or two-way ANOVA followed by the Tukey–Kramer or Newman–Keuls *post hoc* test or with a two-tailed unpaired *t*-test. These statistical tests were selected to be appropriate for the data properties (normality of distribution and homogeneity of variance). A *p*-value less than 0.05 was considered significant. Statistical tests were performed with Minitab 16 software (Minitab Ltd.) and GraphPad Prism 8 (GraphPad Software, San Diego, CA, USA). Extreme outliers were removed using Grubbs’ test provided by GraphPad (GraphPad Software) at *p*-value < 0.05. Survival estimation analysis and KM plot generation were performed using KM Plotter [21] or MedCalc (MedCalc).

## Supplementary information

Supplementary figure legends and the primer sequences

Supplementary Figures and Table

aj-checklist_CDDIS-21-2935R

author-contribution-form_CDDIS-21-2935

## Data Availability

Datasets generated and/or analyzed during the current study are available from the corresponding author on reasonable request.
