# Supplementary material for: Targeting prooxidant MnSOD effect inhibits triple-negative breast cancer (TNBC) progression and M2 macrophage functions under the oncogenic stress
Source: Cell Death Dis. 2022 Jan 11;13(1):49. doi: 10.1038/s41419-021-04486-x (PMC8752602; doi:10.1038/s41419-021-04486-x)
Supplement: Supplementary file 1 — Supplementary figure legends and the primer sequences [file 41419_2021_4486_MOESM1_ESM.docx]

**Supplementary information**

**Targeting prooxidant MnSOD effect inhibits triple-negative breast cancer (TNBC) progression and M2 macrophage functions under the oncogenic stress**

Aushia Tanzih Al Haq^1,2^, Hong-Yu Tseng^1^, Li-Mei Chen^1^, Chien-Chia Wang^2^

and Hsin-Ling Hsu^1*^

^1^Institute of Molecular and Genomic Medicine, National Health Research Institutes, Miaoli, Taiwan

^2^Department of Life Sciences, National Central University, Taoyuan, Taiwan

*Correspondence: hsinling88@nhri.edu.tw

Institute of Molecular and Genomic Medicine, National Health Research Institutes, 35 Keyan Road, Zhunan, Miaoli County 35053, Taiwan.

**This PDF file includes:**

**Supplementary Figure Legends and The Primer Sequences**

| Fig. S1 | *MCTS1*^high^/*NFE2L2*^high^ is implicated in poor clinical outcomes. |
| --- | --- |
| Fig. S2 | MnSOD modulates EMT and breast cancer stemness. |
| Fig. S3 | Differentiation, mobility and phagocytosis of macrophages. |
| Fig. S4 | MCT-1 and IL-6 escalate mROS. |
| Fig. S5 | MCT-1 promotes TNBC cell invasion via mROS. |
| Fig. S6 | Immunohistochemistry of MDA-MB-231 tumors. |

Table S1 The primer sequences

**Supplementary Figure Legends and Primer Sequences**

**Figure S1. *MCTS1*^high^/*NFE2L2*^high^ is implicated in poor clinical outcomes. (A)** Quantitative RT-PCR validated *NFE2L2* mRNA levels in MCF-10A and MDA-MB-231 cells with or without MCT-1 overexpression (control vs. MCT-1). *ACTB* (β-actin) was used as an internal control. Data are presented as the mean ± s.e.m. **(B)** KM Plotter analysis was used to evaluate the relapse-free survival of breast cancer patients stratified by low (n=1661) versus high *NFE2L2* (n=1524) levels. **(C)** The correlation of *MCTS1* and *NFE2L2* expression in breast cancer patients (n=1949) was analyzed using the Pawitan dataset in the Oncomine cancer microarray database. Statistical analysis was performed using a two-tailed unpaired Student’s *t*-test **(A)**, the log-rank Mantel-Cox test **(B)** or the Pearson product-moment correlation coefficient **(C).**

**Figure S2**. **MnSOD modulates EMT and breast cancer stemness. (A)** Metastatic MDA-MB-231 cells without or with MCT-1 overexpression (control vs. MCT-1) and MnSOD depletion (scramble vs. shMnSOD, #7 and #8) were studied. GAPDH was used an internal control. **(B)** MDA-MB-231 cell migration was measured using a wound closure assay under different MCT-1 and MnSOD expression conditions. **(C)** Representative gelatin zymography and quantification of MMP-9 and MMP-2 secreted by MDA-MB-231 cells in different MCT-1 and MnSOD expression backgrounds. **(C)** Cancer stem cell markers (*EPCAM, NANOG, SOX2, SNAI1, PROM1* and *ALDH1A1*) were assayed in MDA-MB-231 mammospheres (day 14) under different MCT-1 and MnSOD expression conditions. *ACTB* was used as an internal control. **(E)** MDA-MB-231 (scramble vs. shMCT-1) cells with vehicle transfection (MOCK) or MnSOD overexpression (Myc-DDK-MnSOD) were studied. Immunoblotting results confirmed the transfectants. **(F)** The transformation of these transfected cells was evaluated by a soft agar colony formation assay. Scale bar, 75 µm. **(G)** Mammospheres (≥ 50 µm in diameter) generated by cells with different MCT-1 and MnSOD expression contexts were analyzed. Scale bar, 50 µm. **(H)** Representative flow cytometry plots (left) and quantification (right) of the CD44(+)/CD24(-) population in mammospheres with or without enriched MnSOD (day 11). Data are presented as the mean ± s.e.m. Statistical analysis was performed using two-way analysis of variance (ANOVA) followed by Tukey-Kramer *post hoc* analysis. *p<0.05; **p<0.01; ***p<0.001.

**Figure S3**. **Differentiation, migration and phagocytosis of macrophages. (A)** Schematic diagram showing the differentiation of THP-1 monocytes into M0, M1, and M2 macrophages by stimulation with phorbol 12-myristate 13-acetate PMA (100 nM) for 48 hr, followed by treatment with lipopolysaccharides (LPS) (100 ng/ml) or IL-4 (20 ng/ml) and IL-13 (20 ng/ml) for another 48 hr. **(B-C)** After THP-1 monocytes were treated with or without PMA, the pan-macrophage marker *ADGRE1* (F4/80) was quantified by qRT-PCR **(B)** and Western blotting **(C)**. *ACTB* was used as an internal control. **(D)** Flow cytometric analysis was used to assess F4/80 expression on PMA-induced THP-1 macrophages. **(E-F)** The mRNA (*CD163* and *MRC1*) **(e)** and protein levels (Arginase-1 and IL-10) **(F)** of M2 macrophage markers were examined after M0 THP-1 macrophages cocultured with MDA-MB-231 cells with MnSOD knockdown and/or MCT-1 overexpression for 48 hr in a Transwell chamber. **(G)** The migratory abilities of M0 (n=16), M1 (n=13), and M2 (n=20) THP-1 macrophages were examined using a single-cell tracking assay. Images were recorded at three different positions for 24 hr at 10-min intervals. Macrophage trajectories emanating from the origin (left) were plotted using the DiPer program. **(H)** Experimental design and gating strategy for *in vitro* phagocytosis of GFP(+) MDA-MB-231 cells by CTFR(+) M1 THP-1 macrophages. Phagocytosis was assessed as the frequency of GFP(+)/CTFR(+) events out of all events. **(I)** Flow cytometry-based phagocytosis assay for GFP(+) MDA-MB-231 cells engulfed by CTFR(+) THP-1 macrophages (M0, M1 and M2). **(J)** Phagocytosis of CTFR(+) 4T1 cells by CD11b(+) RAW264.7 macrophages (M0, M1 and M2). Data are presented as the mean ± s.e.m. Statistical analysis was performed using a two-tailed unpaired Student’s *t*-test **(B, D)** and two-way analysis of variance (ANOVA) **(E)** or one-way analysis of variance (ANOVA) **(G, I, J)** followed by Tukey-Kramer *post hoc* analysis *p<0.05; **p<0.01; ***p<0.001.

**Figure S4**. **MCT-1 and IL-6 escalate mROS. (A)** Quantification of mROS in response to IL-6 stimulation of MDA-MB-231 cells (control vs. MCT-1). **(B-C)** Mitochondrial ROS levels in MDA-MB-231 cells (control vs. MCT-1) were compared upon diphenyleneiodonium (DPI) **(B)** or Rotenone (Rot) **(C)** exposure. Quantification was performed using the fluorescent probe MitoSOX and flow cytometric analysis. Data are presented as the mean ± s.e.m. Statistical analysis was performed using two-way analysis of variance (ANOVA) followed by Tukey-Kramer *post hoc* analysis. **p<0.01; ***p<0.001.

**Figure S5**. **MCT-1 promotes TNBC cell invasion via mROS. (A-D)** Quantification of mROS levels and invasiveness of MDA-MB-231 **(A-B)** and HCC1395 **(C-D)** cells upon exposure to Rotenone (Rot) and in combination with MitoQ. MitoSOX was used to quantify mROS levels **(A, C)**. Invasiveness was assayed in a Transwell invasion chamber **(B, D)**. Representative invasive cells stained with crystal violet are shown (left) and quantified (right). Data are presented as the mean ± s.e.m. Statistical analysis was performed using two-way analysis of variance (ANOVA) followed by Tukey-Kramer *post hoc* analysis **(A-D)**. **p<0.01; ***p<0.001.

**Figure S6**. **Immunohistochemistry of MDA-MB-231 tumors.** Representative images at 5x magnification of MDA-MB-231 tumors immunohistochemically stained with an anti-CD163 Ab and counterstained with hematoxylin and eosin (H&E). Scale bar, 500 µm.

**Supplementary Table S1**.

**Table S1**. The primer sequences for the study.
