# Supplementary material for: Targeting prooxidant MnSOD effect inhibits triple-negative breast cancer (TNBC) progression and M2 macrophage functions under the oncogenic stress
Source: Cell Death Dis. 2022 Jan 11;13(1):49. doi: 10.1038/s41419-021-04486-x (PMC8752602; doi:10.1038/s41419-021-04486-x)
Supplement: Supplementary file 2 — Supplementary Figures and Table [file 41419_2021_4486_MOESM2_ESM.pdf]

Supplementary Figures

Fig. S1

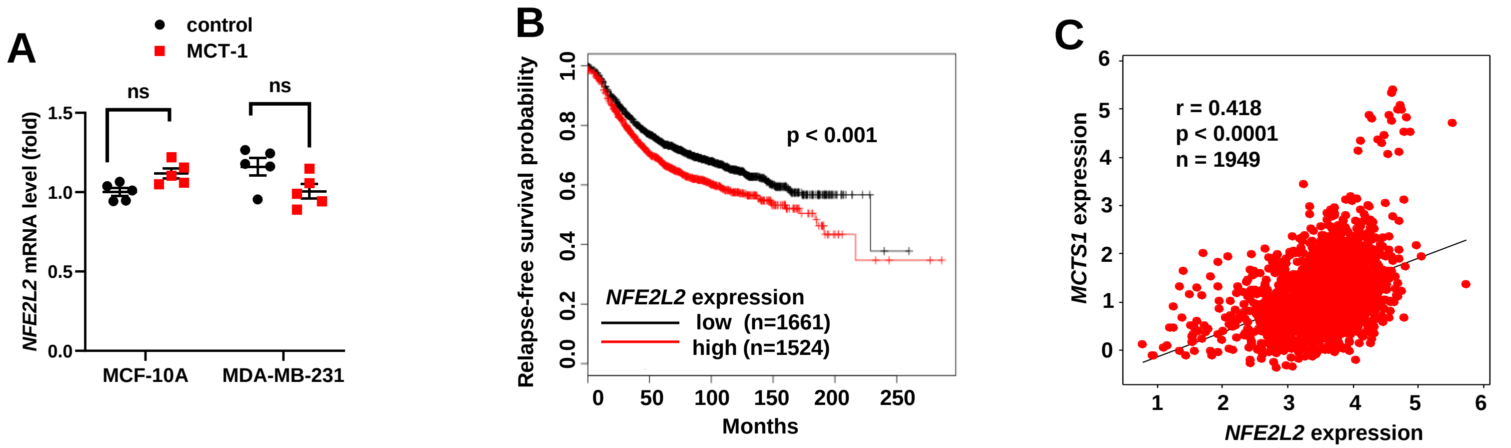

A
control
MCT-1
ns
ns
1.5
1.0
0.5
0.0
NFE2L2 mRNA level (fold)
MCF-10A
MDA-MB-231
B
Relapse-free survival probability
1.0
0.8
0.6
0.4
0.2
0.0
p < 0.001
NFE2L2 expression
low (n=1661)
high (n=1524)
0
50
100
150
200
250
Months
C
r = 0.418
p < 0.0001
n = 1949
MCTS1 expression
6
5
4
3
2
1
0
1
2
3
4
5
6
NFE2L2 expression

Fig. S2

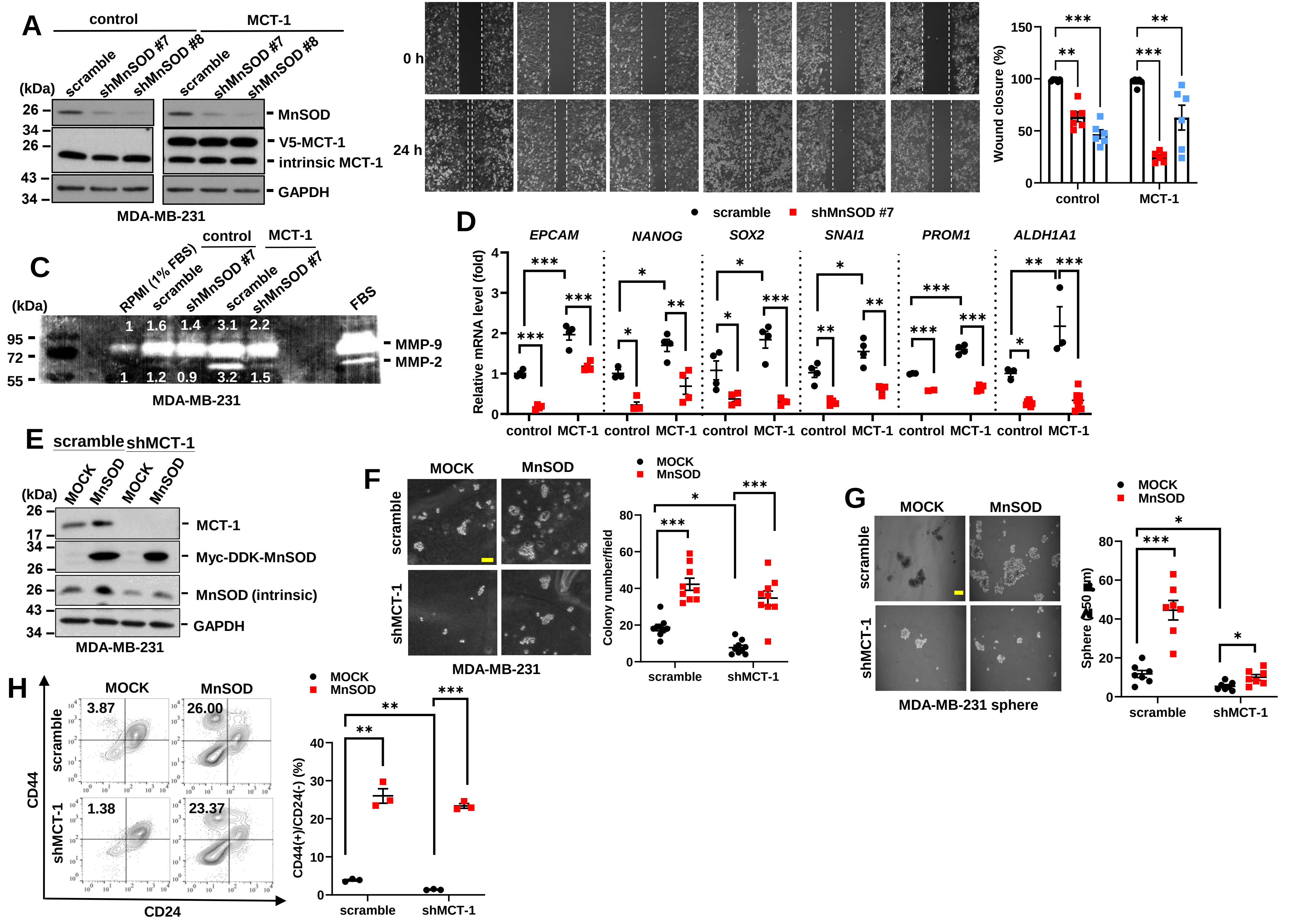
A
control
MCT-1
scramble
shMnSOD #7
shMnSOD #8
(kDa)
26
34
26
43
34
MnSOD
V5-MCT-1
intrinsic MCT-1
GAPDH
MDA-MB-231
B
control
MCT-1
scramble
shMnSOD #7
shMnSOD #8
0 h
24 h
Wound closure (%)
150
100
50
0
control
MCT-1
C
(kDa)
RPMI (1% FBS)
FBS
95
72
55
1
1.6
1.4
3.1
2.2
1.2
0.9
3.2
1.5
MMP-9
MMP-2
MDA-MB-231
D
scramble
shMnSOD #7
EPCAM
NANOG
SOX2
SNAI1
PROM1
ALDH1A1
Relative mRNA level (fold)
4
3
2
1
0
E
scramble
shMCT-1
MOCK
MnSOD
17
MCT-1
Myc-DDK-MnSOD
MnSOD (intrinsic)
F
shMCT-1
Colony number/field
80
60
40
20
G
MDA-MB-231 sphere
H
3.87
26.00
1.38
23.37
CD44
CD24
CD44(+)/CD24(-) (%)
30
10

Fig. S3

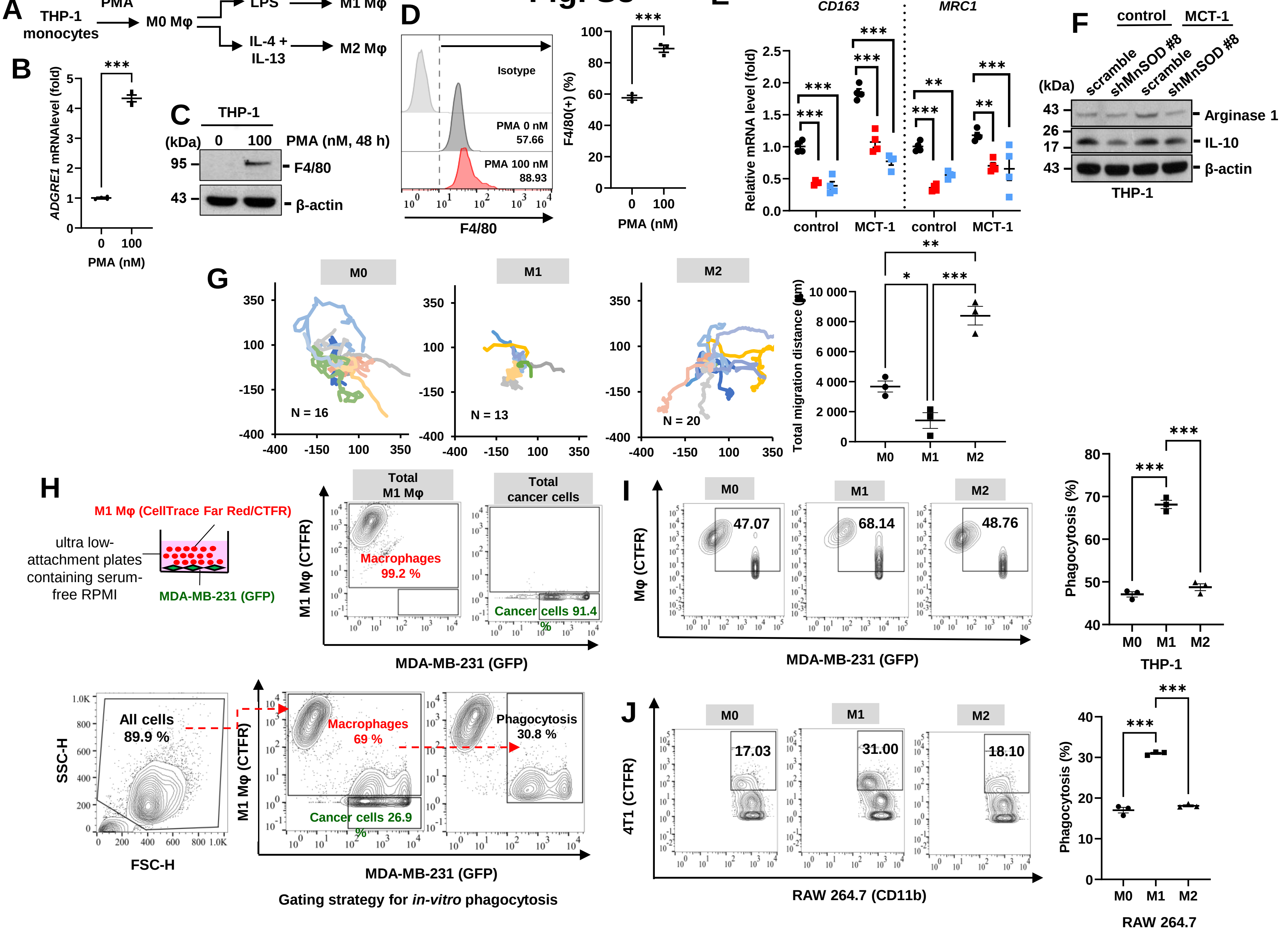

A
THP-1 monocytes
PMA
M0 Mφ
LPS
M1 Mφ
IL-4 + IL-13
M2 Mφ
B
ADGRE1 mRNAlevel (fold)
PMA (nM)
C
THP-1
(kDa)
PMA (nM, 48 h)
F4/80
β-actin
D
Isotype
PMA 0 nM
57.66
PMA 100 nM
88.93
F4/80
F4/80(+) (%)
PMA (nM)
E
scramble
shMnSOD #7
shMnSOD #8
CD163
MRC1
Relative mRNA level (fold)
control
MCT-1
F
control
MCT-1
scramble
shMnSOD #8
(kDa)
Arginase 1
IL-10
β-actin
THP-1
G
M0
M1
M2
N = 16
N = 13
N = 20
Total migration distance (μm)
H
M1 Mφ (CellTrace Far Red/CTFR)
ultra low-attachment plates containing serum-free RPMI
MDA-MB-231 (GFP)
Total M1 Mφ
Total cancer cells
M1 Mφ (CTFR)
Macrophages 99.2 %
Cancer cells 91.4 %
All cells 89.9 %
SSC-H
FSC-H
Macrophages 69 %
Cancer cells 26.9 %
Phagocytosis 30.8 %
Gating strategy for in-vitro phagocytosis
I
Mφ (CTFR)
47.07
68.14
48.76
Phagocytosis (%)
THP-1
J
4T1 (CTFR)
17.03
31.00
18.10
RAW 264.7 (CD11b)
RAW 264.7

Fig. S4

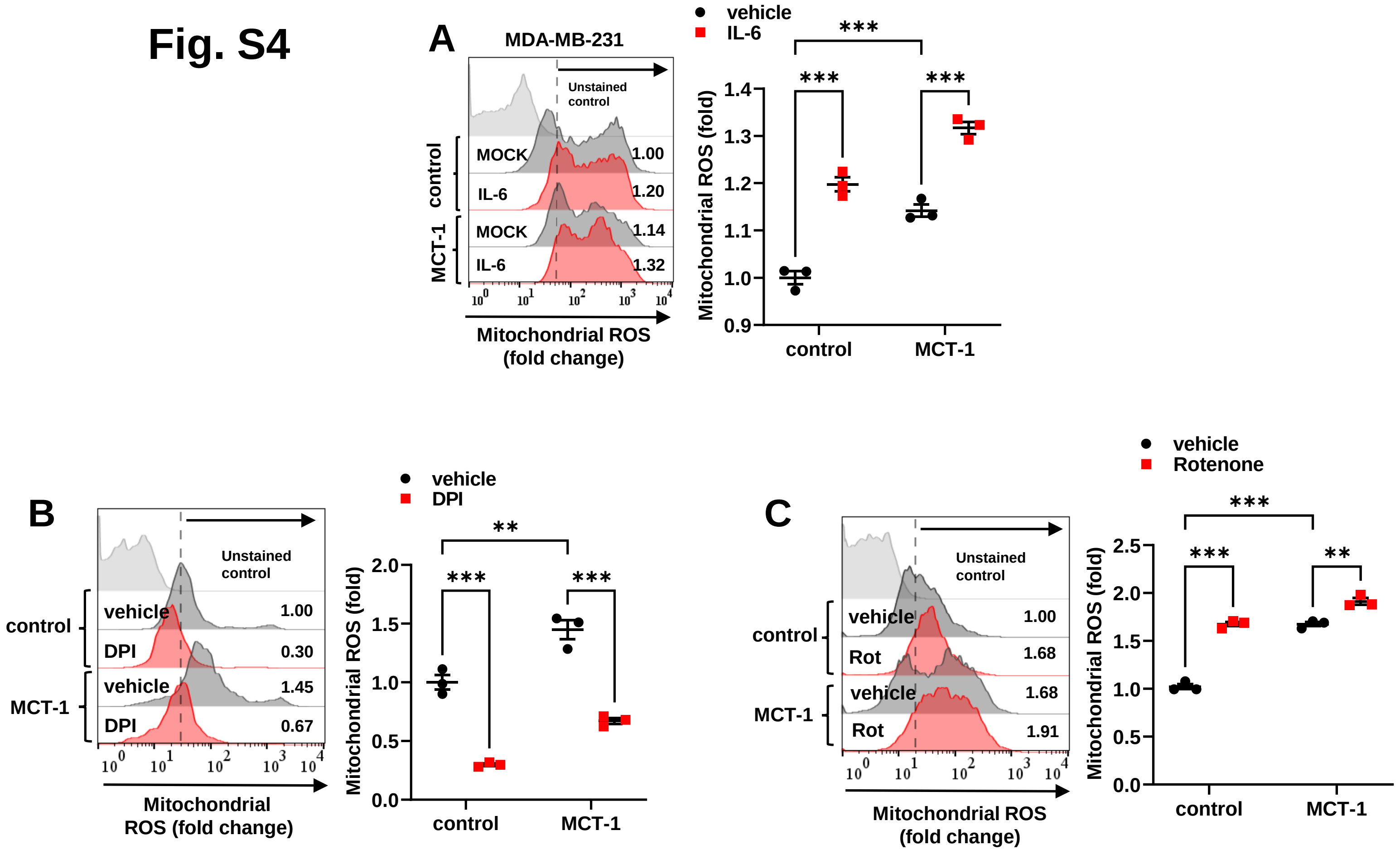
A
MDA-MB-231
Unstained control
control
MOCK 1.00
IL-6 1.20
MCT-1
MOCK 1.14
IL-6 1.32
Mitochondrial ROS (fold change)
vehicle
IL-6
Mitochondrial ROS (fold)
control
MCT-1
B
Unstained control
control
vehicle 1.00
DPI 0.30
MCT-1
vehicle 1.45
DPI 0.67
Mitochondrial ROS (fold change)
vehicle
DPI
Mitochondrial ROS (fold)
control
MCT-1
C
Unstained control
control
vehicle 1.00
Rot 1.68
MCT-1
vehicle 1.68
Rot 1.91
Mitochondrial ROS (fold change)
vehicle
Rotenone
Mitochondrial ROS (fold)
control
MCT-1

Fig. S5

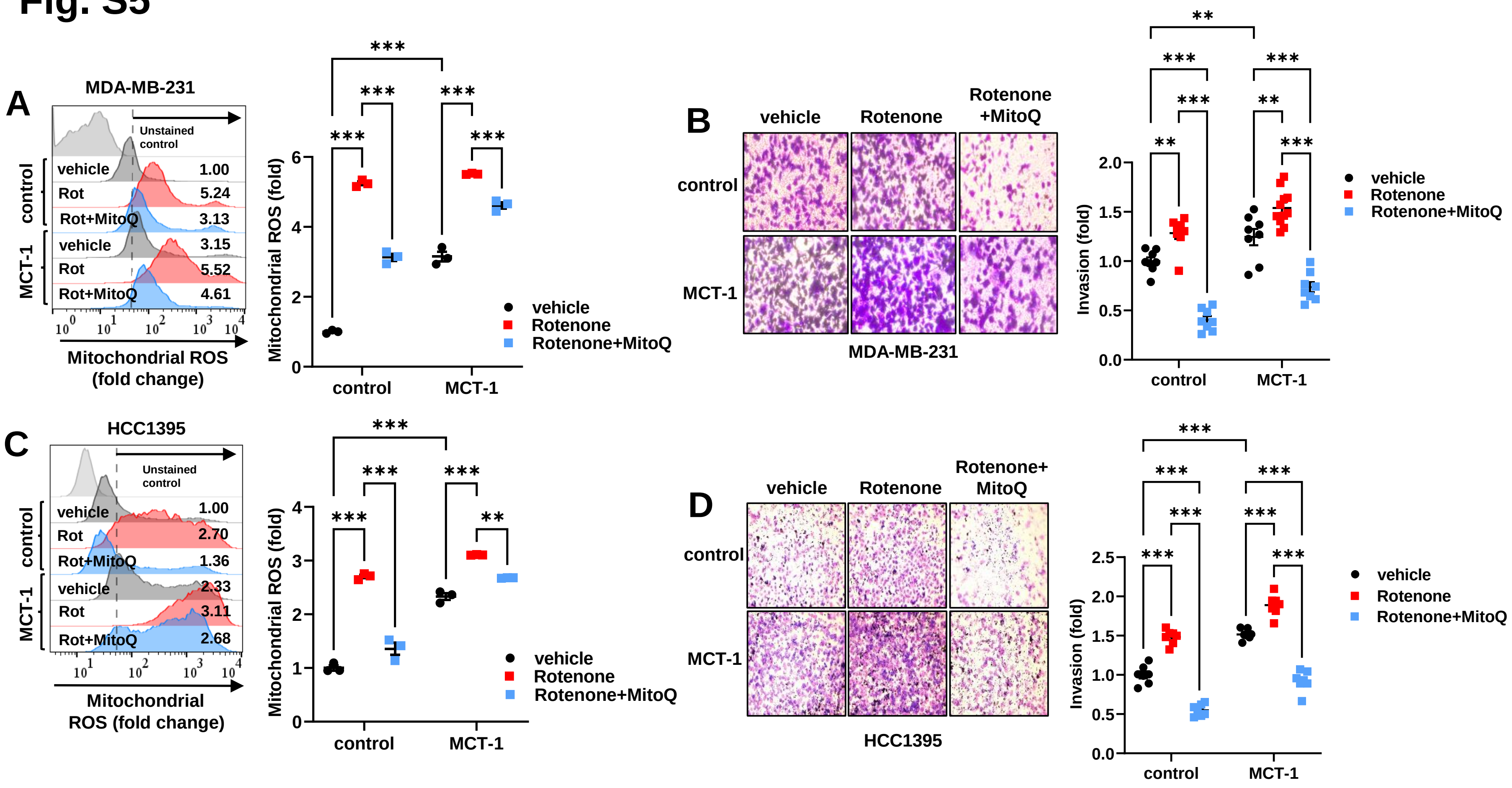

A
MDA-MB-231
Unstained control
vehicle 1.00
Rot 5.24
Rot+MitoQ 3.13
vehicle 3.15
Rot 5.52
Rot+MitoQ 4.61
control
MCT-1
Mitochondrial ROS (fold change)
Mitochondrial ROS (fold)
vehicle
Rotenone
Rotenone+MitoQ
B
vehicle
Rotenone
Rotenone +MitoQ
control
MCT-1
MDA-MB-231
Invasion (fold)
C
HCC1395
Unstained control
vehicle 1.00
Rot 2.70
Rot+MitoQ 1.36
vehicle 2.33
Rot 3.11
Rot+MitoQ 2.68
Mitochondrial ROS (fold change)
D
Rotenone+ MitoQ
HCC1395

Fig. S6

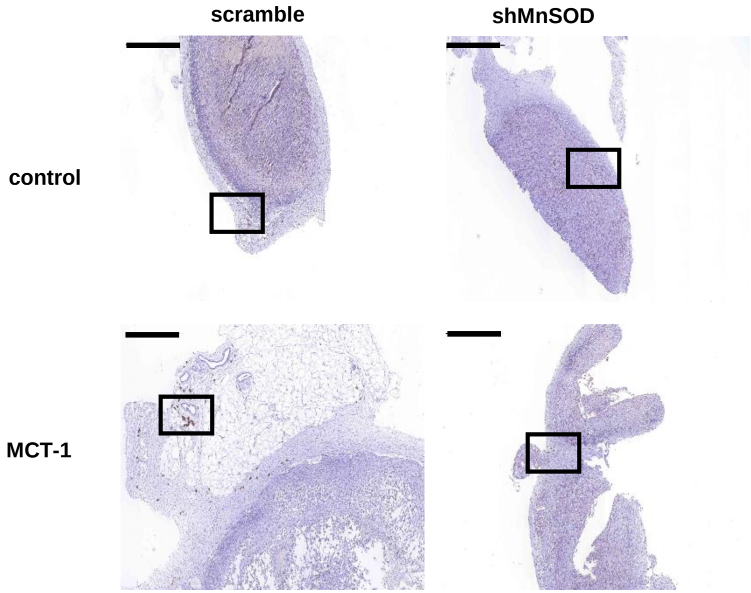

Supplementary Table S1

Primer sequences		
SOD2 (MnSOD)	Forward	5′-AGCTATTTGGAATGTAATCAACTGG-3′
	Reverse	5′-TAAGCAACAT CAAGAAATGCTACA-3′
NFE2L2 (Nrf2)	Forward	5′-TCTGCCAACTACTCCCAGGT-3′
	Reverse	5′-AGTGACTGAAACGTAGCCGAA-3′
ADGRE1 (F4/80)	Forward	5’-CAATGAGTGCCTCACCAGCA-3’
	Reverse	5’-TGGGCAAGCTCTTGGATCTG-3’
CD163	Forward	5′-CCG GGAGATGAATTCTTGCCT-3′
	Reverse	5′-AGACACAGAAATTAGTTCAGCAGCA-3′
MRC1 (CD206)	Forward	5’-CTGAATTGTACTGGTCTGTCCT-3’
	Reverse	5’-GCTTAGATGTGGTGCTGTGG-3’
ALDH1A1	Forward	5’-CACGCCAGACCTACCTGTCC-3’
	Reverse	5’-GCA GAG CTCCTCAGT TG-3’
CD44	Forward	5’-TTACAGCCTCAGCAGAGCAC-3’
	Reverse	5’-TGACCTAAGACGGAG GGAGG-3’
EPCAM	Forward	5’-TGCTGGAATTGTTGTGCTGG-3’
	Reverse	5’-AGATGTCTTCGTCCCACG-3’
SNAI1	Forward	5’-GCGAGCTGCAGGACTCTAAT-3
	Reverse	5’-GGACAGAGTCCCAGATGAGC-3’
SOX2	Forward	5’-CATGAAGGAGCA CCCGGATT-3’’
	Reverse	5’-TTAATGTGCGCG TAACTGTG-3’
PROM1 (CD133)	Forward	5’-GGGAATGCCTACTGGAA-3’
	Reverse	5’-AATTTGCATGAA AGCACAAGG-3’
NANOG	Forward	5’-TGGGAAGAAGCTAAAGAGCCA G-3’
	Reverse	5’-GGA TGCTTCAAAGCAAGG CA-3’
ACTB (β-actin)	Forward	5′-CACCAGGGCGTGATG GTGGG-3′
	Reverse	5′-GATGCCTCTCTTGCTCTGGGC-3′
