# Supplementary material for: Targeting prooxidant MnSOD effect inhibits triple-negative breast cancer (TNBC) progression and M2 macrophage functions under the oncogenic stress
Source: Cell Death Dis. 2022 Jan 11;13(1):49. doi: 10.1038/s41419-021-04486-x (PMC8752602; doi:10.1038/s41419-021-04486-x)
Supplement: Supplementary file 4 — author-contribution-form_CDDIS-21-2935 [file 41419_2021_4486_MOESM4_ESM.pdf]

DECLARATION OF CONTRIBUTIONS TO ARTICLE

ADMC

Manuscript Number: CDDIS-21-2935

Journal Name: *Cell Death & Disease* (the 'Journal')

Proposed Title of the Contribution: Targeting prooxidant MnSOD effect inhibits triple-negative breast cancer (TNBC) progression and M2 macrophage functions under the oncogenic stress (the 'Contribution)

Author(s): Aushia Tanzih Al Haq, Hong-Yu Tseng, Li-Mei Chen, Chien-Chia Wang, and Hsin-Ling Hsu (the 'Authors')

For all *CDDis* articles, each person named as an author in the published version must be able to show he or she has contributed substantially to the article.

Authorship credit should be based on 1) substantial contributions to conception and design, acquisition of data, or analysis and interpretation of data; 2) drafting the article or revising it critically for important intellectual content; and 3) final approval of the version to be published. Authors should meet conditions 1, 2 and 3.

Any person who cannot be shown to have made a substantial contribution to the article cannot be listed as an author in the final version. The name of any person who is deemed to have made a minor contribution can, however, appear in the Acknowledgments section of the article.

Please complete the table below to indicate the contributions of all named authors to the manuscript.

Author Full Name:	Specification of Contribution to the Manuscript:
Aushia Tanzih Al Haq	design, data acquisition & analysis, wrote the manuscript
Hong-Yu Tseng	data acquisition & analysis
Li-Mei Chen	data acquisition
Chien-Chia Wang	provided critical feedback, helped supervise the project
Hsin-Ling Hsu	conceptualization, wrote the manuscript, supervised the project

Please complete the table below to indicate the contributions of all named authors to the figures.

Figure 1:

Data mining were performed by A.T.A (Figs. 1A-C, 1G-I and Supplementary Figs. S1B-C). Experiments and data analysis were performed H.Y.T. (Figs. 2D-F) and A.T.A (Supplementary Fig. S1A)

Figure 2:

Experiments and data analysis were performed by A.T.A (Figs. 2A-J and Supplementary Figs. S2A-H) with critical feedback from C.C.W (Fig. 2E).

Figure 3:

Experiments were performed by L.M.C. (Supplementary Fig. S3C) and A.T.A (all others).

Figure 4:

Experiments and data analysis were performed by A.T.A. (Figs. 4A-N and Supplementary Figs. S4A-C).

Figure 5:

Experiments and data analysis were performed by A.T.A. (Figs. 5A-E and Supplementary Figs. S5A-D).

Figure 6:

Animal studies were performed by A.T.A. with help from H.Y.T (Figs. 6A-E and Supplementary Fig. S6). Data mining was performed by A.T.A. (Fig. 6F). Both A.T.A and H.L.H. contributed to the graphical summary (Fig. 6G).

Signed for and on behalf of the Author(s):	Print Name:	Date:
徐欣伶 Hsin-Ling Hsu	Hsin-Ling Hsu	November 22, 2021
